# Cyclophosphamide- and doxorubicin-induced impairment of high affinity choline uptake and spatial memory can be prevented by dietary choline supplementation in breast tumor bearing mice

**DOI:** 10.1371/journal.pone.0305365

**Published:** 2024-11-21

**Authors:** Robert Botelho, Cheryl L. Kirstein, Rex M. Philpot

**Affiliations:** 1 Department of Psychiatry and Behavioral Neurosciences, Morsani College of Medicine, University of South Florida, Tampa, Florida, United States of America; 2 Department of Molecular Pharmacology and Physiology, Morsani College of Medicine, University of South Florida, Tampa, Florida, United States of America; 3 Department of Psychology, USF College of Arts & Sciences, University of South Florida, Tampa, Florida, United States of America; Nelson Mandela African Institute of Science and Technology, UNITED REPUBLIC OF TANZANIA

## Abstract

AC chemotherapy (Adriamycin and Cytoxan, i.e., doxorubicin and cyclophosphamide, respectively), a common treatment for breast cancer, can lead to significant cognitive side effects, known as Chemotherapy-Related Cognitive Impairments (CRCIs). These cognitive impairments can persist over 20 years and significantly affect the quality of life for cancer patients and survivors. AC chemotherapy is known to impair ovarian function and reduce circulating estradiol (E^2^), an effect that can decrease high-affinity choline uptake (HACU) and reduce acetylcholine (ACh) availability. Because ACh is involved in attention, learning and memory function we hypothesized that the cognitive deficits observed during and after adjuvant chemotherapy (AC) are associated with compromised high affinity choline uptake (HACU) due to suppressed ovarian function. Increasing available choline has been demonstrated to enhance HACU under conditions of demand for ACh, therefore we propose that choline supplementation can mitigate CRCIs by maintaining cholinergic function throughout and following chemotherapy treatment. Our study demonstrates cognitive deficits in tumor-bearing but not non-tumor-bearing mice during and following AC chemotherapy, suggesting that tumors enhance vulnerability to CRCIs. We found that HACU was impaired in tumor-bearing mice administered AC chemotherapy and that a choline-enriched diet can mitigate both the reduction of HACU induced by chemotherapy and deficits in spatial memory, suggesting a protective role of dietary choline against disruptions in HACU and cognitive impairment caused by chemotherapy. This underscores the potential use of dietary choline supplementation as a part of chemotherapeutic interventions.

## Introduction

Breast cancer is the second leading cause of death in the United States, affecting 13% of women in their lifetime. Fortunately, there is a large population of breast cancer survivors due to an overall 5-year survival rate of 91% [1, 2]. AC chemotherapy is an effective treatment intervention that utilizes doxorubicin (DOX; Adriamycin) and cyclophosphamide (CYP; Cytoxan) and is highly recommended in the treatment of breast cancer at all stages [3–7]. While these agents are effective in killing cancerous cells, the side effects of AC chemotherapy can significantly impact the quality of life (QOL) of patients and survivors. Chemotherapy-Related Cognitive Impairments (CRCIs) are a significant side effect of chemotherapy that affects up to 75% of chemotherapy patients [8, 9] and can persist for 20+ years after treatment [10–13]. CRCIs encompass a range of learning and memory impairments, including deficits in spatial memory, attention, concentration, information processing speed, verbal and visual memory, and executive function [10–19]. Not surprisingly, the development of CRCIs has become a significant concern for those who require chemotherapy. The frequency of breast cancer diagnoses and the potential enduring cognitive side effects of AC chemotherapy point to a need for an increased understanding of the mechanisms that underlie CRCIs and the development of treatment strategies that can reduce their occurrence.

Several chemotherapeutic agents, including AC chemotherapy, reduce circulating estradiol (E^2^) due to ovarian suppression or ablation [5, 20–23]. E^2^ may act as an allosteric modulator of the CHT1 transporter [24–26], which is critical for high-affinity choline uptake (HACU). Indeed, both *in vitro* and *in vivo* studies have shown that increasing available E^2^ accelerates HACU [24, 27]. HACU is the rate-limiting step in the synthesis of acetylcholine (ACh) [28] and as a consequence, chemotherapy-induced reductions in circulating E^2^, and subsequently HACU, will lead to reductions in ACh synthesis and availability. A reduction in ACh is significant for cognitive function as cholinergic signaling is involved in attention, encoding, learning, and verbal, visual, and spatial memory [29–35], cognitive domains affected in CRCIs. Indeed, impairments in the cholinergic system have been implicated in CRCI development [11, 36–41]. Therefore, chemotherapy-mediated impairment of circulating E^2^ and HACU may contribute to CRCIs.

Hormone Replacement Therapy (HRT) could potentially restore circulating E^2^ to address reductions in HACU. However, for breast cancer patients, HRT is not advisable due to an increased risk of recurrence [42]. An alternative approach involves increasing dietary choline. Studies indicate that acute injections of choline chloride or iodide can prevent choline depletion in key brain regions such as the cerebral cortex, striatum (STR) or hippocampus (HCC) under conditions demanding enhanced ACh production [43–45]. Further, acute injection of choline chloride, or increasing choline chloride concentrations in the diet, can increase HACU under conditions of demand for ACh [33, 46–48]. [43–45] Our previous work in Balb/C mice has shown that a 2% choline supplementation can mitigate spatial memory deficits induced by AC chemotherapy [49]. We propose that increased choline availability protects brain function by maintaining normal cholinergic activity despite disruptions caused by chemotherapy.

In the context of cancer, elevated dietary choline has not been associated with an increased risk of recurrence and is linked to a lower incidence of breast cancer and reduced cancer mortality [50]. However, given the elevated choline levels in tumors and cancer’s characterization as a choline metabolism disorder, there is a concern that increased choline availability could potentially promote tumor growth or reduce chemotherapy effectiveness. To address these concerns, our study employed both non-tumor bearing mice and the MMTV-PyVT mouse model of breast cancer [51]. We investigated how increased choline intake affects tumor development, growth and the efficacy of CYP+DOX treatment.

We hypothesize that the cognitive deficits observed during and following AC chemotherapy may be linked to compromised HACU resulting from the suppression of ovarian function. We suggest that choline supplementation can counteract these cognitive deficits by sustaining cholinergic function during and after chemotherapy treatment. Our experimental approach using both non-tumor (NTB) and tumor-bearing (TB) female mice involved several key investigations: 1) we determined whether CYP+DOX administration affects ovarian function; 2) we examined the impact of CYP+DOX on HACU in the frontal cortex (fCTX), STR and HCC–regions crucial for working and spatial memory; 3) we evaluated how CYP+DOX influences spatial memory in MMTV-PyVT mice; 4) we determine whether 2% dietary choline can prevent CYP+DOX-induced deficits in HACU and spatial memory; and lastly, we assessed the implications of 2% choline diet for tumor development and growth and its impact on the effectiveness of CYP+DOX.

## Methods

All procedures involving animals were approved by the Institutional Animal Care and Use Committee and performed in accordance with the institution’s guidelines [52]. Using relevant data in the literature [35, 53–55] and preliminary studies to estimate effect size, the sample size necessary to achieve a statistical power of 0.80 for each experiment was determined using G*Power software (Christian-Albrechts-University, Kiel, Germany), resulting in 9–12 animals per group for each portion of the study.

*B6*.*FVB-Tg* (MMTV-PyVT) *634Mul/LellJ* mice were bred in-house using hemizygous male mice and *C57BL/6J* female mice. Breeders and their offspring were given free access to water and standard Teklad Global 18% protein rodent chow (TD.2018, Envigo) containing 0.12% choline. A total of 477 female MMTV-PyVT mice were utilized for different components of these studies. Animal group assignments as well as attrition results are outlined in S1 Fig.

### Genotyping

On PND 21, when in-house bred MMTV-PyVT mice were weaned, a tissue sample from the tail of each female mouse was collected and genotyped (Transnetyx, Cordova, TN) as MMTV-PyVT: Tg negative (NTB; N = 253) or positive (TB; N = 224). Female mice were subsequently housed by genotype, 3–4 per cage. Male mice were euthanized via CO^2^ inhalation followed by cervical dislocation.

### Timeline, weights, tumor assessment and measurement

**Fig 1:**
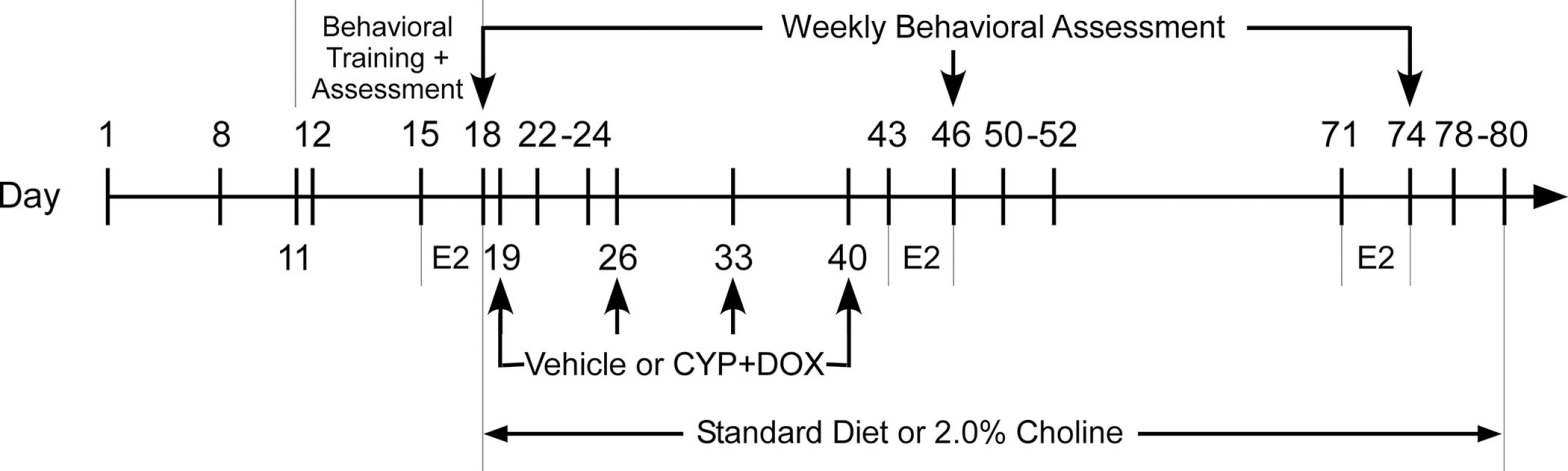
Study timeline and methodological overview. Female mice were monitored weekly for tumor development through palpation and external measurement. Day 1 indicates the first day of the week of tumor identification. Following baseline MWM testing, mice were assigned to groups on Day 8, followed by orientation and training in the Morris Water Maze (MWM) from Days 11 to 18 to evaluate spatial memory. Following baseline MWM testing Mice either continued on a standard diet or transitioned to a 2% choline diet on Day 18. Assessment of proestrus status and submandibular blood draws were conducted on Days 15–18, 43–46, and 71–74. Mice received weekly injections of either a combination of cyclophosphamide (CYP; 66.7 mg/kg, i.v.) and doxorubicin (DOX; 6.7 mg/kg, i.v.) or saline on Days 19, 26, 33, and 40. Subsequent evaluations of spatial memory were conducted on Days 46 and 74 to assess the impact of chemotherapy. Mice were euthanized via decapitation and tissue was collected for high-affinity choline uptake (HACU) assays between Days 22–24, 50–52, and 78–80.

Female mice were weighed and monitored weekly for tumor manifestation via manual manipulation and external tumor measurement using calipers. TB mice were assigned to a cohort upon detection of a tumor greater than 50 mm^3^ (using the modified ellipsoidal formula: Volume_(tumor)_ = ½(Length x Width^2^). The age of tumor emergence was between 88 and 126 days. We have verified that our volume measurements have a strong positive correlation with postmortem tumor weights (r = 0.86, p< 0.05), indicating it is a valid estimate of tumor mass in our hands. The first day of the week of tumor detection was designated as Day 1 (Fig 1).

On Day 8, mice were randomly assigned to remain on the standard diet (TD.2018, Envigo) or be transitioned to a 2% choline diet on Day 18 (TD.150428, Envigo) immediately following baseline testing in the Morris water maze, and were housed according to dietary assignment. Tumor-bearing cohorts assigned to each diet were group housed, 3–4 per cage with age-matched non-tumor-bearing mice of the same cohort housed separately, 3–4 per cage. To track tumor development and progression weekly examinations of tumor-bearing mice were conducted to record the number of palpable tumors and measure the volume of each using calipers.

### Morris Water Maze (MWM)

The MWM was used to assess the acquisition and retention of spatial memory as described in Philpot et. al. [35]. This behavioral task was chosen based on published studies in both mice and humans indicating that chemotherapeutic agents significantly impair the performance of females in this task [35, 54–57].

The apparatus consisted of a circular pool (130 cm in diameter, 60 cm in height) filled with water made opaque by adding non-toxic paint. The water was maintained at a constant temperature of 22–24°C. Mice were trained to locate a clear Plexiglas platform hidden beneath the water’s surface using visual cues located outside of the water tank.

The pool was divided into 6 equal size ‘virtual’ zones using EthoVision XT tracking software, which was used to record and analyze behavior in the maze. This included the escape latency (time taken to find the platform), swimming speed, swimming distance, and time and distance moved in the arena and individual zones.

#### MWM orientation/ training/ testing

Orientation occurred on Days 11–12. The orientation phase’s purpose was to acclimatize the mice to the water and to teach them that active searching will lead to the platform location. During the orientation phase, each mouse was introduced to the pool from varying starting points and the platform was placed 3 mm above the surface of the water at varying endpoints. A Plexiglas rod, extending 10 cm vertically, was placed on the platform to aid in platform location. Each mouse received 5 trials a day for 2 days before beginning training.

Training occurred on Days 13–17. During the training phase, the platform was hidden 2 mm below the water’s surface. The platform location was varied across all 6 zones, but remained constant for individual mice so they could learn its location relative to cues in the environment. Mice received 5 trials per day for 5 days. On every training day, each mouse was introduced into the pool from each of the 5 starting zones that did not contain the platform. For each trial, mice were given an opportunity to locate the hidden platform for a maximum of 60 sec. If the mouse did not find the platform, it was gently guided towards it and allowed to remain on the platform for 15 sec to facilitate spatial orientation. After the trial mice were removed from the pool, dried gently, and placed in a holding cage warmed by a heating pad to prevent hypothermia.

Testing occurred on Days 18, 46 and 74. On testing days, trials 1, 2, 4 and 5 all occurred as described during training. However, on trial 3 (Probe Trial) the platform was removed from the pool and the mouse allowed to search for 60sec after being placed in a random, non-platform zone. The proportion of the distance moved in the zone which previously contained the platform relative to the total distance moved during the probe trial (Probe Trial Exploration) was used to indicate active searching for the platform in the correct location. Mice that did not produce at least 20% of their movement in the zone that previously contained the platform during baseline testing were removed from further study. The addition of 2 platform trials following the probe trial on each testing day served to reduce extinction effects that can be introduced by probe trial testing.

### Proestrus measurement and submandibular blood draws

Proestrus was assessed in each mouse using an electronic vaginal-estrous cycle monitor (Stoelting Co., Wood Dale, IL) on Days 15–18, 43–46, and 71–74, and submandibular blood drawn for analysis of circulating E^2^. Proestrus was indicated by an impedance measurement of ≥ 4 kΩ [58]. Submandibular blood draws occurred on the 1^st^ day that proestrus was detected in each mouse and, if proestrus was not detected on any of the 4 days of measurement, blood draws occurred on day 4 following behavioral assessment. We elected to assess mice over a 4-day period because the typical estrus cycle of mice occurs over a period of 4–5 days and thus 4 consecutive days of measurement should produce at least 1 proestrus measurement from most mice in a normal estrus cycle. Data analyzed from the proestrus measurements from each mouse was whether there was an impedance measurement of ≥ 4 kΩ on at least 1 of the 4 days at each of the 3 time points.

### Diet

On Day 18, following baseline MWM testing, mice were placed on a 2% choline diet or remained on a standard diet (TD.2018, Envigo) depending on their group assignment and housing. Established research indicates that a 2% choline diet enhances free choline in circulation by over 50% and elevates free choline concentrations in hippocampal and striatal sections of animals on this diet [33]. Consequently, it is plausible that choline-mediated increases in free choline may compensate for alterations in the function of cholinergic projections to the hippocampus following the administration of chemotherapeutic agents. The 2% choline diet consisted of 977.8 g of TD.2018 and 22.2 g of choline chloride (TD.150428, Envigo; [59]). The standard TD.2018 and 2% choline TD.150428 diets were 18.6 or 17.8% protein, 6.2 or 5.7% fat and 44.2 or 46.9% carbohydrate, respectively. Both diets provided 3.1 kcal/g.

Because mice were group housed and pellets of the 2% choline diet were prone to crumbling, it was not assumed that monitoring of the frequency and amount of diet provided per cage could accurately reflect the intake of individual mice, and therefore regular measurements of the diet were not taken.

### Chemotherapy

CYP was obtained from Tocris Bioscience and dissolved in 0.9% saline (20 mg/mL). 0.9% saline was made in-house. DOX (2 mg/mL) was obtained from Teva Parenteral Medicines.

Following baseline assessment in the MWM (Day 18), mice that demonstrated spatial memory of the platform location by devoting ≥ 20% of their Day 18 probe trial exploration (total distance swam) to the zone that contained the platform during training, were randomly assigned to receive weekly injections via the tail vein of either CYP (66.7 mg/kg, i.v.) + DOX (6.7 mg/kg, i.v.) or equivalent volumes of saline (Days 19, 26, 33 and 40). The doses used for this study correspond to a cumulative dose of 800 and 80 mg/m^2^ for CYP and DOX, respectively, doses in the 4-week cumulative dose range for standard breast cancer chemotherapy, with the LD-50 reported in the literature as 140 mg/kg for cyclophosphamide [60] and 10 mg/kg for doxorubicin [61]. Our preliminary data indicates that this dose regimen attenuates tumor growth and impairs spatial memory in MMTV-PyVT mice. On days 46 and 74, spatial memory was reassessed in the MWM to assess the effect of chemotherapy on spatial memory and the persistence of these effects, respectively.

### Blood/ tissue collection for analysis

Twelve mice per group (N = 96) were sacrificed on days 22–24 (3–5 days following 1 injection), 50–52 (10–12 days following 4 injections), or 78–80 (38–40 days following the final injection). Tissue samples from the fCTX, STR, and HCC were harvested for analysis of HACU. Trunk blood samples were collected on those days and centrifuged for 15 min (1960 rcf) at 4⁰ C, then the serum was collected and stored at -80⁰ C for E^2^ analysis.

### HACU assays

Regional tissue samples were homogenized in 0.32 M sucrose and centrifuged at 1160 rcf (4⁰ C) for 10 min to remove large cellular debris and nuclei. Subsequently, the supernatant was collected and spun at 20,800 rcf (4⁰ C) for 20 min to produce a pellet of isolated synaptosomes. The supernatant from this second spin was poured off and the pellet was re-suspended in 0.32 M sucrose (ratio 1:80) for HACU and protein assays.

For each sample, we prepared 2 tubes containing an incubation solution of 0.1 mL 1 nmol choline per 0.5 uCi of [3^H^] choline and 0.8 mL Krebs-Ringers HEPES buffer. One tube of each sample was placed in a water bath (37⁰ C) and the other was placed on ice (0⁰ C). A volume of 0.1 mL of each synaptosomal fraction was added to the hot and cold incubation tubes and vortexed to ensure even distribution. After 5 minutes of incubation, the contents of the hot and cold tubes were vacuum filtered through 0.45 uM pore filter paper, the filter paper was placed into 20 mL scintillation vials and dissolved with 1 mL glycol monoethyl ether (Fisher) on a shaker (15–30 minutes). A volume of 10 mL of scintillation fluid (Ecolume, MP Biomedicals, Solon, OH) was added to each vial, vortexed, and counted on a Beckman LS 6500 scintillation counter.

### Statistical analysis

For all statistical analyses, an *a priori* p-value of 0.05 was used to define statistical significance. For multiple comparisons, Sidak’s correction for family-wise error was used. A 4 factor 2 (Tumor Status) X 2 (Diet) X 2 (Treatment) X 12 (Day) ANOVA was performed on weekly weights while 3 factor 2 (Diet) X 2 (Treatment) X 12 (Day) ANOVA was performed on tumor number and volume and weight-adjusted total tumor volume. For survival analysis, Chi-square was performed on the frequencies of deaths across weeks of assessment for each tumor-bearing group with subsequent chi-square comparisons to isolate effects. Chi-square analysis was also performed to assess significant changes in the frequency of proestrus across Tumor Status, Diet, Treatment, and Weeks of assessment. A 4 factor 2 (Tumor Status) X 2 (Diet) X 2 (Treatment) X 3 (Week) ANOVA was performed on circulating E^2^ concentrations. For MWM training a 3 factor 2 (Tumor Status) X 3 (Training Day) X 5 (Trial) ANOVA was conducted on total swim distance during the platform trials to assess acquisition of an effective platform search strategy. For MWM performance on probe trials, the proportion of movement in each zone was analyzed using a 5-factor 2 (Tumor Status) X 2 (Diet) X 2 (Treatment) X 3 (Week) X Zone (6) mixed factorial ANOVA. For HACU results a 2 (Tumor Status) X 2 (Diet) X 2 (Treatment) X 3 (Week) X 3 (Brain Region) MANOVA was performed to assess overall effects. Subsequent univariate 2 (Tumor Status) X 2 (Diet) X 2 (Treatment) X 3 (Week) ANOVA we conducted within each brain region to isolate effects.

## Results and discussion

### Weights

**Fig 2:**
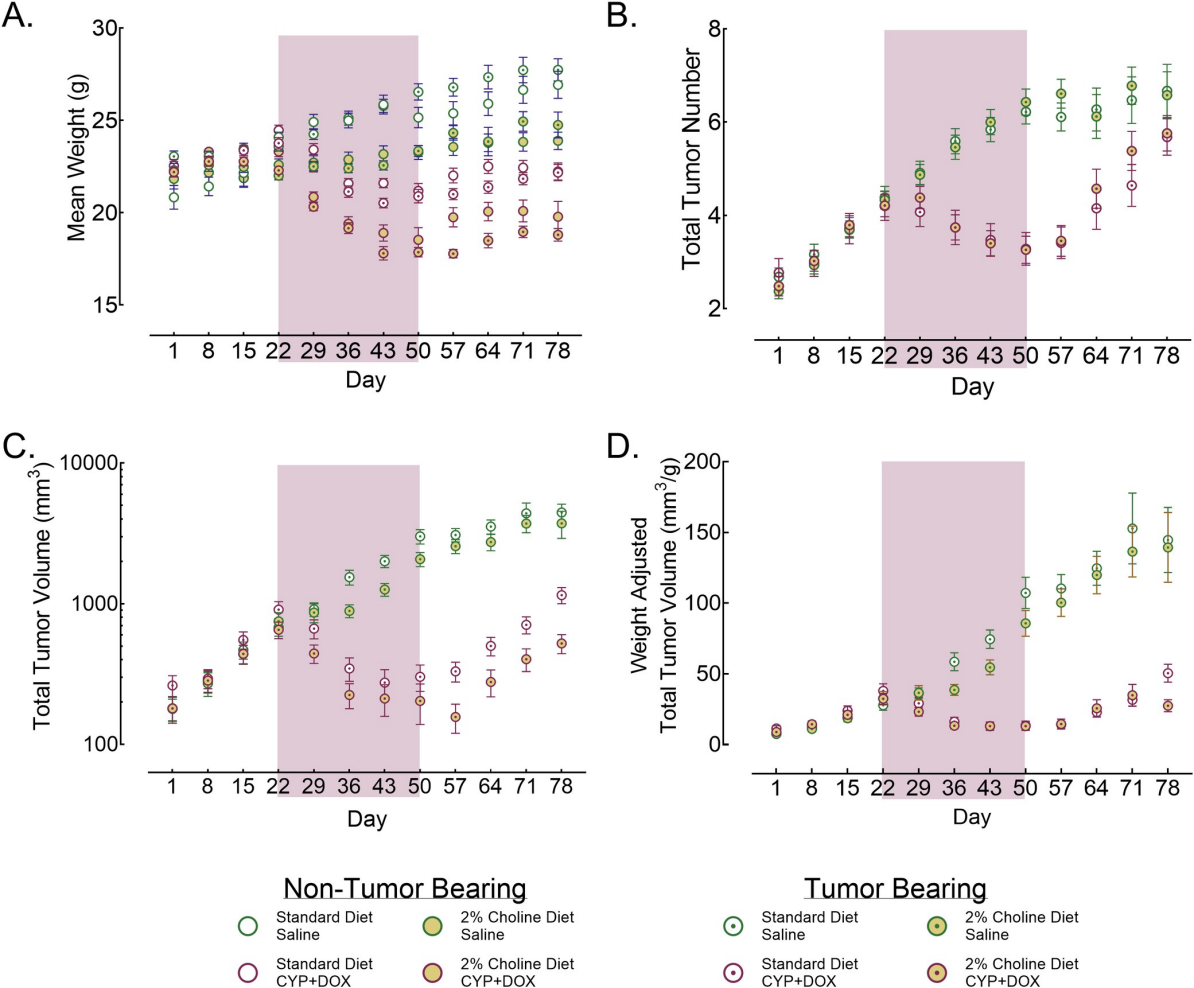
Longitudinal assessment of animal weights and tumor metrics. For panels A-D, values represent group means +/- S.E.M.; the shaded red area indicates week 1 through week 4 of cyclophosphamide (CYP; 66.7 mg/kg, i.v.) and doxorubicin (DOX; 6.7 mg/kg, i.v.) or saline injections; Color and symbol references for groups are in the legend. Initial group sizes for non-tumor bearing mice: standard diet saline injected (n = 81); standard diet CYP+DOX- injected (n = 69); 2% choline diet saline injected (n = 54); 2% choline diet CYP+DOX- injected (n = 49). Initial group sizes for tumor bearing mice: standard diet saline injected (n = 58); standard diet CYP+DOX-injected (n = 49); 2% choline diet saline injected (n = 64); 2% choline diet CYP+DOX-injected (n = 53). For all comparisons, a p<0.05 after Sidak’s correction for family-wise error was considered significant. A) Weights of mice in each experimental group throughout the study. This panel illustrates the impact of 2% choline, introduced on Day 18, and weekly injections on Days 19, 26, 33, and 40 of CYP+DOX on weight. B) Number of detectable tumors in tumor-bearing mice, reflecting tumor incidence and proliferation under different treatment conditions. C) Total tumor volume of mice, reflecting the summed volumes of all tumors per mouse to demonstrate the growth dynamics of tumors in response to the experimental interventions. D) Total tumor volume normalized by animal weight to account for the influence of body mass on tumor size.

Weights of both tumor-bearing (TB) and age-matched non-tumor-bearing (NTB) mice were recorded weekly (Fig 2A) beginning the week tumors first emerged (Day 1). The results of a factorial ANOVA indicate significant differences in weight associated with interactions between Tumor Status and Chemotherapy [F (11, 2789) = 12.81, p<0.05], Diet and Timepoint [F (11, 2789) = 7.36, p<0.05], and Chemotherapy and Timepoint [F (11, 2789) = 42.06, p<0.05]. When assigned to a cohort (Day 1), the weights of NTB and TB mice did not differ significantly (21.5 +/- 0.43 g and 22.4 +/- 0.16 g, respectively). Additionally, across the course of the study (Day 1 through Day 78), NTB and TB mice receiving saline injections had similar average weights (24.2 +/- 0.12 g and 23.8 +/- 0.8 g, respectively). However, mice receiving CYP+DOX injections weighed significantly less than mice administered saline (21.4 +/- 0.09 g and 24.2 +/- 0.07 g, respectively) throughout the study. Similarly, CYP+DOX-treated TB mice weighed significantly less than their NTB counterparts (21.2 +/-0.09 g vs 22.0 +/- 0.13 g, respectively).

The weights of mice assigned to either the Standard diet or 2% Choline diet groups were not significantly different from Day 1 to Day 15 (22.5 +/- 0.2 g STD; 23.3 +/- 0.2 g 2% choline) while they remained on the standard diet. However, 4 days after being placed on a choline diet (Day 22), mice in the 2% choline group weighed 6.6% less than mice on a standard diet [F (1, 357) = 8.96, p<0.05]. Over the following 8 weeks (Day 22–78), 2% choline animal weights ranged between 20.9 +/- 0.24 g and 22.6 +/- 0.37 g, consistently less than standard diet mice (range: 23.5 +/- 0.22 g to 24.7 +/- 0.37 g). These data demonstrate attenuated weight gain after mice are switched to a choline diet.

The weights of mice assigned to receive Saline or CYP+DOX injections were not significantly different in the weeks before receiving injections, from Day 1 to Day 22. However, following one injection of CYP+DOX, on Day 29, CYP+DOX mice weighed significantly less (7.2%) than their saline counterparts. This pattern continued across the weeks of injections, with CYP+DOX weights decreasing approximately 0.5 g/week over the following 3 weeks (20.5 +/- 0.18 g on Day 36 to 19.6 +/- 0.23 g on Day 50). These weights were significantly lower than the saline group, whose weights increased approximately 0.6 g/week over the same period (23.9 +/- 0.18 g on Day 34 to 24.7 +/- 0.23 g on Day 50). From Days 57–78 CYP+DOX injected mice experienced weight gain (range: 20.0 +/- 0.24 g to 21.5 +/- 0.32 g), however, the weights of these mice remained significantly lower than saline-injected mice (range: 25.0 +/- 0.26 g to 25.6 +/- 0.36 g). These data demonstrate that CYP+DOX can attenuate weight gain in mice leading to weight differences between treatment groups that persists for at least 35 days following CYP+DOX exposure.

### Tumor number

Factorial ANOVA results indicated a significant effect of CYP+DOX injections on tumor emergence in TB mice from Days 1 to 78 (Fig 2B; [F (11,1908) = 18.69, p<0.05]). Initially, from Days 1 to 22, both the saline and CYP+DOX treated groups exhibited a similar increase in tumor numbers, with increases ranging from 12–24% per week. However, from Days 29–50, saline-injected mice experienced a weekly tumor number increase of 7–13%, while CYP+DOX-injected mice showed smaller increases of 0 and 10% per week. The difference in the rate of tumor emergence resulted in significantly fewer tumors in CYP+DOX injected mice than saline-injected mice during the weeks of AC chemotherapy and highlights the effectiveness of CYP+DOX at attenuating tumor emergence.

After the cessation of CYP+DOX treatment (Days 57–78), the pattern of reduced tumor emergence in CYP+DOX-treated mice relative to saline-treated mice reversed. The saline group’s rate of tumor emergence slowed, changing by -4 to 3% per week. By contrast, the CYP+DOX group saw a substantial increase in tumor numbers, with numbers increasing by 4–27% per week. Despite this increased rate of emergence, the CYP+DOX group maintained a significantly lower tumor number compared to the saline group for the first 3 weeks following the end of chemotherapy. However, by Day 78 the tumor counts in both groups reached similar levels.

The observed slowing of tumor number increases in the saline group might be due to the merging of growing tumors, complicating counting accuracy. Conversely, the increase in tumor numbers in the CYP+DOX group post-treatment can be attributed to the smaller size of individual tumors, reducing the likelihood of merging and allowing emerging tumors to be accurately counted.

### Tumor volume

Factorial ANOVA revealed significant reductions in tumor volume in mice treated with CYP+DOX [F (11,1909) = 51.50, p<0.5]. During the initial phase of the study, from Days 1 to 22, we noted a substantial increase in total tumor volume, ranging from 12% to 88% per week (Fig 2C). From Days 29–50, saline-injected mice continued to exhibit an increase in tumor volume of 30% and 69% per week. By contrast, CYP+DOX-injected mice exhibited a notable decrease in tumor volume during this time, ranging from -7 to -47% per week. This reduction was consistent regardless of the animals’ diet, underscoring the effectiveness of CYP+DOX in reducing tumor volume.

Following the cessation of CYP+DOX injections (Days 57–78), tumor growth in the CYP+DOX injected mice increased by 6% to 63% per week while tumor volume changed by -7% to 24% per week among saline-injected mice. Nevertheless, tumor volumes in the CYP+DOX group remained significantly smaller than those in the saline group. The observed slowing of tumor volume growth rates in the saline group during the final weeks (Days 57–78) could be attributed to the merging of tumors, which complicates accurate volume measurements across weeks.

Saline-injected mice on a standard diet had larger average tumor volumes across the course of the study (Days 1 to 78) compared to those on choline supplemented diet (1597 +/- 88 mm^3^ vs. 1306 +/- 73 mm^3^, respectively; [F (1,1909) = 7.21, p<0.05]). This difference was most pronounced from days 36–57, with Saline + 2% choline mice exhibiting 31–42% smaller tumor volumes than Saline/Standard diet mice. This trend continued throughout the study, suggesting that a 2% choline diet alone may slow tumor growth.

Considering the significant weight reduction in mice on a 2% choline diet, we normalized tumor volumes to account for individual weight differences (Fig 2D). After this adjustment, the tumor volumes of mice in the Saline + Standard diet group remained significantly larger than mice in the Saline + 2% choline group [F(1,1880) = 8.511, p<0.05], indicating that the group difference in tumor volumes was not due to diet associated differences in animal mass. This further confirms the tumor-reducing effect of the 2% choline diet.

### Survival analysis

Survival analysis of TB mice indicated a significant difference between treatment groups in the probability of death due to tumor growth or unknown causes across weeks of assessment [χ^2^ (3) = 13.07, p < 0.05] (S2 and S3 Figs). Among TB mice on a Standard Diet, those receiving CYP+DOX injections were less likely to die across weeks of assessment than Saline injected TB mice [χ^2^ (1) = 7.98, p < 0.05] indicating the practical effectiveness of AC chemotherapy in this mouse model (S3A Fig). Among TB mice on 2% Choline Diet, the probability of death did not differ between mice receiving Saline or CYP+DOX injections [χ^2^ (1) = 3.24, p > 0.05] (S3B Fig). Further, TB mice on 2% Choline Diet were not significantly more likely to die across weeks of assessment than those on Standard Diet whether they received Saline injections [χ^2^ (1) = 1.00, p > 0.05] or DOX+CYP [χ^2^ (1) = 3.13, p > 0.05] (S3C and S3D Fig). This indicates that the 2% Choline diet did not increase the probability of death due to tumor growth or unknown causes and, when considered in conjunction with tumor growth measures, may provide unique benefits when combined with AC chemotherapy.

### Effect of Tumor Status, Diet, and CYP+DOX on proestrus

**Fig 3:**
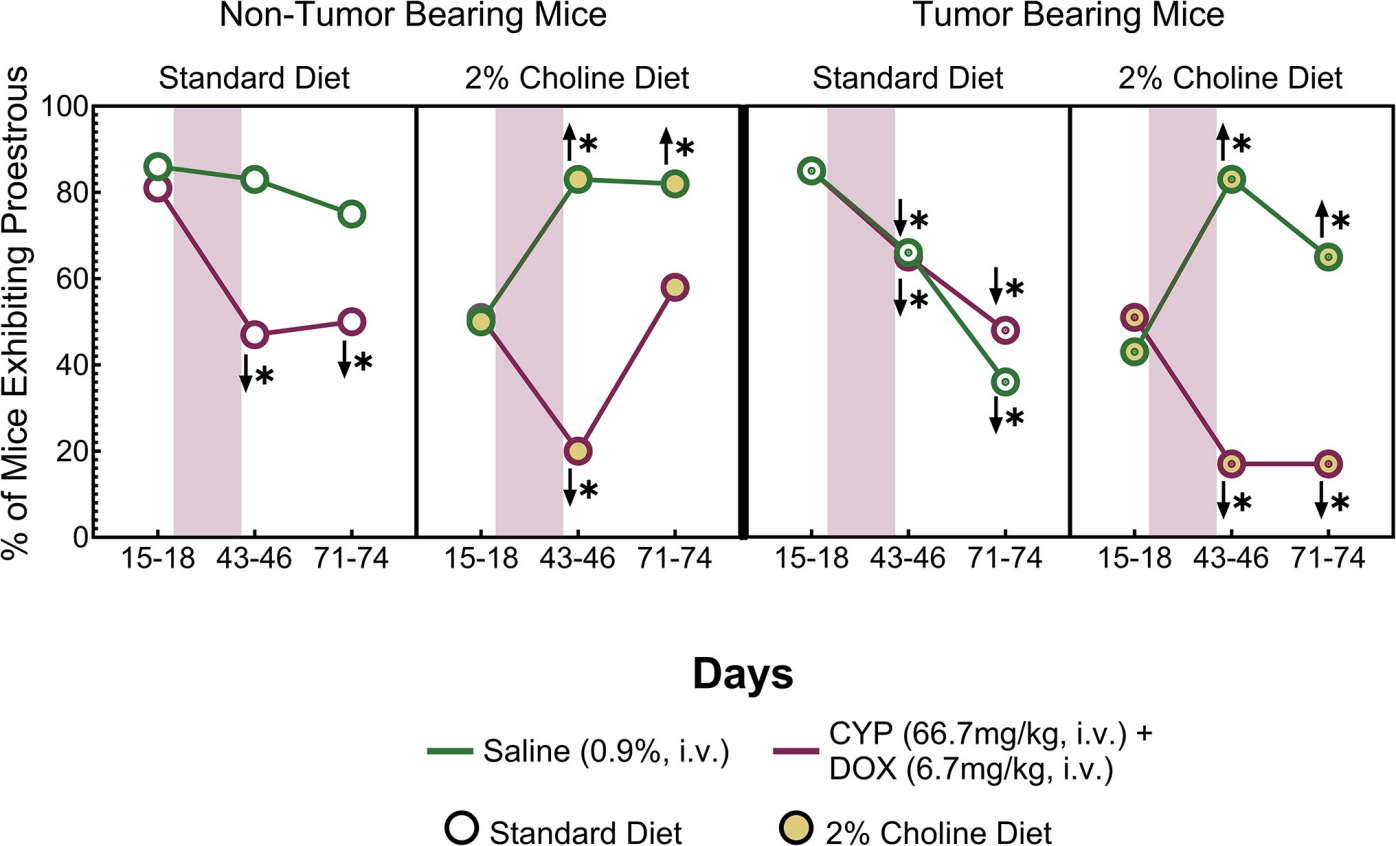
Frequency of proestrus in non-tumor and tumor-bearing female mice. The percentage of female mice exhibiting proestrus [an impedance measurement of ≥ 4 kΩ on at least 1 of the 4 consecutive days of measurement] at baseline (15–18 days), four weeks of chemotherapy (43–46 days), and post-chemotherapy (71–74 days). Initial group sizes for non-tumor bearing mice: standard diet saline injected (n = 81); standard diet CYP+DOX- injected (n = 69); 2% choline diet saline injected (n = 54); 2% choline diet CYP+DOX- injected (n = 49). Initial group sizes for tumor bearing mice: standard diet saline injected (n = 58); standard diet CYP+DOX-injected (n = 49); 2% choline diet saline injected (n = 64); 2% choline diet CYP+DOX-injected (n = 53). The shaded red area indicates week 1 through week 4 of cyclophosphamide (CYP; 66.7 mg/kg, i.v.) and doxorubicin (DOX; 6.7 mg/kg, i.v.) or saline injections. Color and symbol references for groups are in the legend. Arrows indicate significant changes from the baseline within each group, where asterisks denote significant differences between groups at the same timepoint. For all comparisons, a p<0.05 after Sidak’s correction for family-wise error was considered significant.

We examined the frequency of proestrus on Days 15–18, 43–46, and 71–74, and determined its relationship to tumor status, diet, and treatment via Chi-square analysis (Fig 3). At baseline (Days 15–18), for NTB and TB mice on a standard diet, proestrus frequency over 4 days of assessment was similar for mice scheduled to receive saline or CYP+DOX injections, between 83% and 86%. However, NTB and TB mice on a 2% choline diet exhibited proestrus frequencies of 50% to 55% at baseline, significantly lower than mice on a standard diet [X^2^ (6, N = 474) = 39.02, p<0.05].

The administration of CYP+DOX had a significant impact on proestrus frequency. NTB mice treated with CYP+DOX showed a marked reduction in proestrus on days 43–46 and 71–74 when on the standard diet. NTB mice on the choline diet also exhibited a reduction in proestrus frequency on days 43–46. However, these values returned to baseline values for NTB mice on the choline diet by days 71–74. For TB mice on a standard diet receiving either saline or CYP+DOX, there was a significant reduction in proestrus occurrence after 4 weekly injections as well as at the end of the study. However, for TB mice on a choline diet, a different pattern emerged depending on the treatment group. TB mice on a choline diet that received saline injections exhibited an increase in proestrus frequency relative to baseline measurements. By contrast, TB mice on a choline diet receiving CYP+DOX injections exhibited a significant decrease in proestrus frequency relative to baseline. These observations indicate that 2% choline diet, tumor development, and CYP+DOX exposure each influence proestrus frequency.

E^2^

We attempted to link changes in estrus cycling to circulating E^2^ concentrations, however, samples were pooled for analysis due to insufficient individual serum volumes, preventing the determination of individual relationships. At baseline, circulating E^2^ from pooled samples was within the normal range (28–36 pg/ml). Despite the observed changes in proestrus frequency, E^2^ levels following 4 weekly injections of saline or CYP+DOX and at the end of the study were not affected by tumor status, diet, or treatment.

### Morris Water Maze

#### Training

Factorial ANOVA of total swim distance on each training trial (Days 13–17) revealed that mice progressively decreased the swim distance required to locate the hidden platform across trials and days of training [F (16,3760) = 2.11, p<0.05] (S4 Fig). This effect did not differ based on group assignment (diet or chemotherapy) or tumor status. Thus, mice were able to acquire an effective search strategy to locate the platform and the rates of acquisition were similar between NTB and TB mice, suggesting similar learning and spatial memory.

#### Baseline

**Fig 4:**
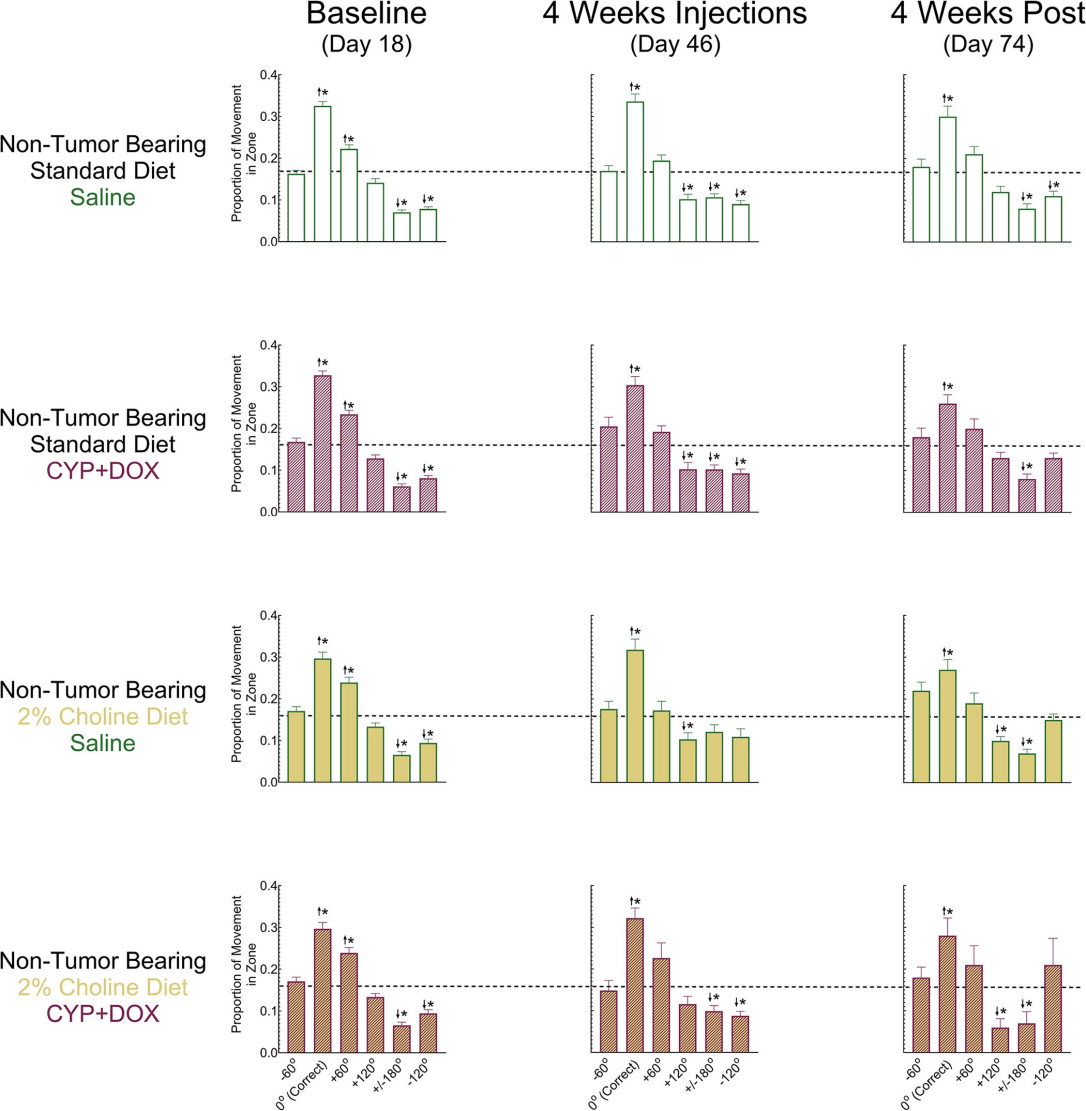
Spatial exploration patterns of non-tumor bearing mice under different treatment conditions in the Morris Water Maze (MWM). The proportion of distance moved for non-tumor bearing mice exploring each of six designated zones in the MWM during the probe trial. Bars represent group means +/- S.E.M.; Dashed line represents chance or unbiased performance (16.67%); 0° corresponds to the target zone where the platform was located during training and platform trials. Each row represents one of the four experimental groups—based on diet (standard or 2% choline) and treatment (saline or CYP+DOX)—across three key time points: baseline (Day 18), after four weeks of treatment (Day 46), and post-chemotherapy (Day 74). Group sizes for standard diet saline injected mice (Day 18 n = 81; Day 46 n = 61; Day 74 n = 48); standard diet CYP+DOX- injected mice (Day 18 n = 69; Day 46 n = 50; Day 74 n = 38); 2% choline diet saline injected mice (Day 18 n = 54; Day 46 n = 33; Day 74 n = 21); 2% choline diet CYP+DOX- injected mice (Day 18 n = 49; Day 46 n = 37; Day 74 n = 27). Upward arrows indicate a greater increase in exploration of a given zone relative to chance, suggesting targeted search behavior, while downward arrows depict less of the zone than chance/unbiased performance, suggesting avoidance or random search patterns. Asterisks denote statistical significance. For all comparisons, a p<0.05 after Sidak’s correction for family-wise error was considered significant.

**Fig 5:**
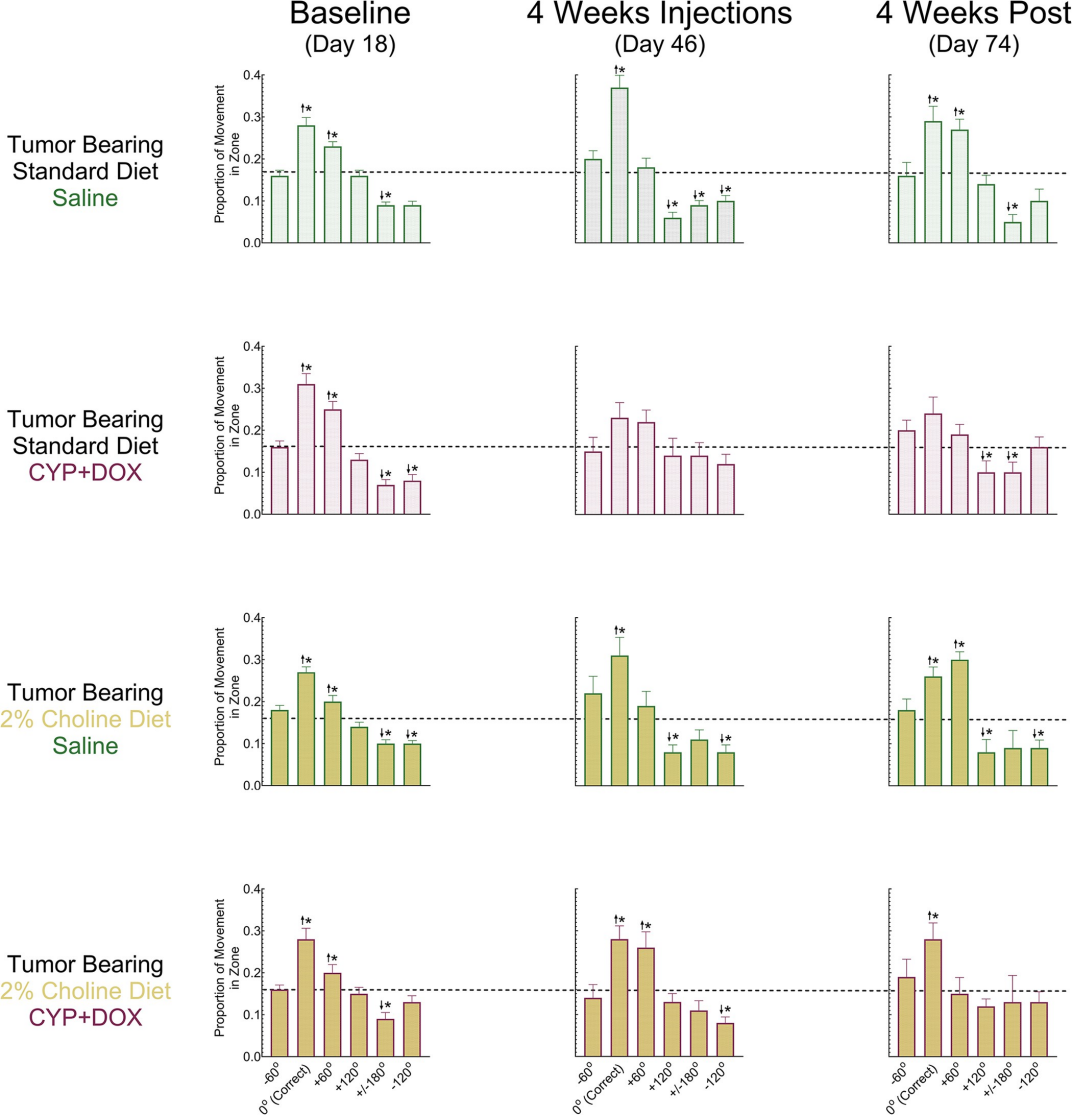
Spatial exploration patterns of tumor bearing mice under different treatment conditions in the Morris Water Maze (MWM). The proportion of distance moved for tumor-bearing mice exploring each of the six designated zones in the MWM during the probe trial. Bars represent group means +/- S.E.M.; Dashed line represents chance or unbiased performance (16.67%); 0° corresponds to the target zone where the platform was located during training and platform trials. Each row represents one of the four experimental groups—based on diet (standard or 2% choline) and treatment (saline or CYP+DOX)—across three key time points: baseline (Day 18), after four weeks of treatment (Day 46), and post-chemotherapy (Day 74). Group sizes for standard diet saline injected mice (Day 18 n = 58; Day 46 n = 45; Day 74 n = 20); standard diet CYP+DOX-injected mice (Day 18 n = 49; Day 46 n = 36; Day 74 n = 23); 2% choline diet saline injected mice (Day 18 n = 64; Day 46 n = 45; Day 74 n = 19); 2% choline diet CYP+DOX-injected mice (Day 18 n = 53; Day 46 n = 40; Day 74 n = 20). 2 Upward arrows indicate greater exploration of a given zone relative to chance, suggesting targeted search behavior, while downward arrows depict less exploration of the zone than chance/unbiased performance suggesting avoidance or random search patterns. Asterisks denote statistical significance. For all comparisons, a p<0.05 after Sidak’s correction for family-wise error was considered significant.

One day following the completion of training (Day 18), all mice were assessed for spatial memory of the platform location using a probe trial. Active exploration of each zone was calculated by dividing the distance moved in each of the 6 individual zones by the total distance moved during the 60-second trial (Figs 4 and 5, for NTB and TB mice, respectively). The calculation of the proportion of movement in each zone was used to control for any differences in overall activity and/or swim velocity that might be associated with the presence of tumors, changes in diet, or exposure to CYP+DOX. Zones were classified as 0° (the zone that contained the platform during training), +/-60° (zones immediately neighboring the platform zone, +/- 120° (zones once removed from the platform zone), or +/-180° (the zone immediately opposite the platform zone).

Repeated measures analysis of the proportion of movement in each of the 6 zones during the baseline probe trial revealed a large main effect of Zone [partial η2 = 0.524] with significant differences in the proportion of exploration of each zone [F(5,1365) = 299.98, p<0.05]. Additionally, significant two-way interactions were observed between Zone and Tumor Status [partial η^2^ = 0.010; F(5,1365) = 2.89, p<0.05], as well as Zone and Diet [partial η^2^ = 0.012; F(5,1365) = 3.45, p<0.05].

At baseline, mice devoted 28–34% of their movement searching the 0° zone, higher than an unbiased behavioral distribution (100% / 6 zones = 16.7%). Although all groups exhibited a preference for the 0° zone during the baseline probe trial, NTB mice devoted significantly more movement exploring the 0° zone (31.6%) than TB mice (28.3%) [F (1,273) = 5.13, p<0.05], and standard diet mice devoted a significantly greater proportion of movement (31.6%) to the 0° zone than their 2% choline diet counterparts (28.5%) [F (1,273) = 5.26, p<0.05]. Additionally, all groups dedicated 22%-27% of their exploration to the zone immediately clockwise (+60°) from the correct zone, indicating a preference for this zone. By contrast, the immediate counterclockwise zone (-60°) was explored at a similar to chance rate by all groups (15–17%).

The proportion of movement in the directly opposing zone (+/- 180°) and its neighboring zones (-120° and +120°), were less than expected if the behavioral distribution was unbiased (16.67%), and ranged between 5% and 15%. Every instance of exploration within the +/- 180° zone was significantly lower than chance (16.7%) indicating certainty that this zone did not contain the platform. Interestingly, the magnitude of this effect differed between NTB and TB mice [F (1,273) = 7.38, p<0.05], with the former devoting 6.8% and the latter 8.8% of exploration to this zone. The +120° zone exploration distance was not significantly different across groups. However, -120° zone exploration demonstrated a significant main effect of Diet [F (1,273) = 11.3, p<0.05], with Standard diet mice devoting a smaller proportion of movement to exploring this zone (8.1%) than their 2% choline counterparts (10.2%). Collectively, these results demonstrate that mice were able to use spatial cues to determine the location of the “correct” (0°) zone and that the groups were similar at baseline.

### Effect of Tumor Status, Diet, and CYP+DOX on spatial memory

After baseline MWM assessment (Day 19), all mice received 4 weekly injections of CYP+DOX or equivalent saline volumes. Spatial memory was reassessed on Day 46, 6 days following the 4^th^ injection, to determine if CYP+DOX exposure impaired the spatial memory of NTB and TB mice and assess whether a 2% choline diet could prevent this impairment. Mice were assessed again on Day 74 to determine whether spatial memory deficits emerged or persisted 34 days following the last injection.

An omnibus mixed factor ANOVA assessing the effect of Tumor Status, Diet, Chemotherapy, and Week of assessment on the proportion of exploration in all zones (6) was performed. This revealed a small (partial η^2^ = 0.02), but significant 4-way interaction of Tumor Status, Diet, Chemotherapy, and Week [F (2,556) = 5.61, p<0.05], as well as a small (partial η^2^ = 0.01), but significant interaction between Tumor Status, Chemotherapy, and Week of Assessment across Zones [F (10,2780) = 2.52, p<0.05].

#### MWM exploratory preference- NTB mice

A large effect (partial η^2^ = 0.39) of Zone on exploratory behavior [F (5,1845) = 232.8, p<0.05] was exhibited by NTB mice during probe trials (Fig 4). NTB mice consistently explored the correct zone (0°) above chance (>16.7%) regardless of Diet or Chemotherapy. Additionally, NTB mice explored the diametrically opposite zone (+/- 180°) at a proportion significantly below chance regardless of Diet or Chemotherapy treatment. This demonstrates the ability of NTB mice to retain the location of the platform and use spatial cues to locate the correct area and indicates that neither CYP+DOX treatment nor a 2% choline diet altered this performance.

A small interaction (partial η^2^ = 0.04) of the Week of assessment on Zone exploratory behavior was also demonstrated in NTB mice [F (10,1845) = 6.66, p<0.05]. Exploratory behavior of NTB mice in the correct zone (0°) remained relatively steady from 31.6% and 32.1% on Days 18 and 46, respectively, decreasing to 28.2% on Day 74 (all significantly above chance; 16.7%), while exploratory behavior in the opposing (+/- 180°) zone increased from 6.8% on Day 18 to 10.7% on Day 46 before decreasing to 7.7% on Day 74 (all significantly below chance; 16.7%).

#### MWM exploratory preference- TB mice

There was a large effect (partial η^2^ = 0.33) of Zone on the exploratory behavior of TB mice [F (5,935) = 90.3, p<0.05] (Fig 5). Additionally, a small (partial η^2^ = 0.04), but significant two-way interaction of Week of assessment and Chemotherapy treatment on exploratory behavior was revealed [F (10,935) = 3.87, p<0.05]. Saline-treated TB mice devoted 27.3% of their exploratory behavior in the correct zone (0°) at baseline, increasing to 34.8% on Day 46 before returning to a baseline value of 27.7% during the final probe trial (Day 74). By contrast, TB CYP+DOX injected mice demonstrated an initial 29.5% exploration preference for the correct zone (0°) on Day 18, which decreased to 24.9% on Day 46, and remained lower on Day 74, at 25.9%. This implies a persistent deficit in the spatial memory of TB mice as a result of CYP+DOX administration.

The effect of CYP+DOX administration on spatial memory in TB mice is due to deficits demonstrated in the standard-diet TB mice, as opposed to 2% choline TB mice. Correct (0°) zone exploration of STD/TB mice at baseline (Day 18) was 31.2%, significantly above chance performance (p<0.05; 16.7%). However, following 4 weeks of CYP+DOX injections (Day 46), correct zone exploration decreased to 22.9% and remained low at 24.5% on Day 74. Performance on Days 46 and 74 was not significantly different from chance performance, implying impaired spatial memory performance in the standard diet TB mouse group.

By comparison, the exploratory behavior in the correct zone (0°) for TB mice on a 2% choline diet was 27.6% on Day 18, significantly greater than chance performance. Following 4 weekly CYP+DOX injections correct zone (0°) exploratory behavior remained significantly greater than chance performance at 27.7% and 27.8% on Days 46 and 74, respectively. These results indicate that TB mice on a 2% choline diet retained the ability to locate the correct zone (0°) following CYP+DOX exposure.

These MWM assessment results demonstrate that the adverse effects of CYP+DOX exposure on spatial memory are unique to TB mice, persist for at least 35 days, and can be attenuated by a 2% choline diet.

### Effect of Tumor Status, Diet, and Chemotherapy on HACU

**Fig 6:**
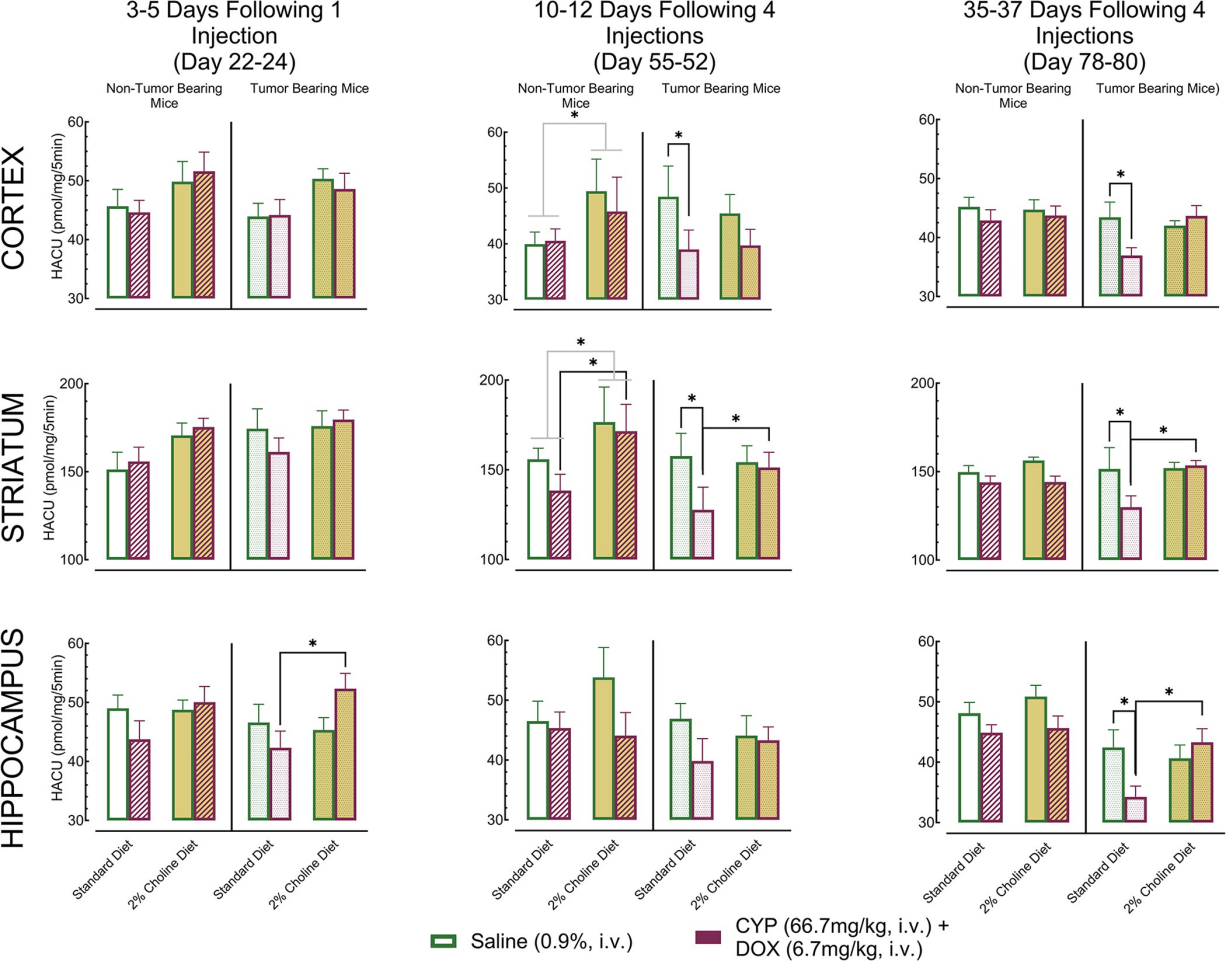
High-affinity choline Uptake (HACU) in the Cortex, Striatum, and Hippocampus of non-tumor and tumor-bearing mice. HACU rates (pmol/mg protein/min) in the frontal cortex, striatum, and hippocampus of non-tumor bearing and tumor-bearing mice. Bars represent group means +/- S.E.M. Each row illustrates HACU in a brain region at different post-injection intervals: 3–5 days following one injection (Day 22–24), 10–12 days following four injections (Day 55–52), and 35–37 days after the last of four injections (Day 78–80). Group sizes for non-tumor bearing mice: standard diet saline injected (Days 22–24 n = 12; Day 55–52 n = 12; Day 78–80 n = 12); standard diet CYP+DOX- injected (Days 22–24 n = 12; Day 55–52 n = 12; Day 78–80 n = 12); 2% choline diet saline injected (Days 22–24 n = 12; Day 55–52 n = 9; Day 78–80 n = 12); 2% choline diet CYP+DOX- injected (Days 22–24 n = 12; Day 55–52 n = 10; Day 78–80 n = 12). Initial group sizes for tumor bearing mice: standard diet saline injected (Days 22–24 n = 12; Day 55–52 n = 11; Day 78–80 n = 9); standard diet CYP+DOX-injected (Days 22–24 n = 12; Day 55–52 n = 9; Day 78–80 n = 12); 2% choline diet saline injected (Days 22–24 n = 12; Day 55–52 n = 12; Day 78–80 n = 12); 2% choline diet CYP+DOX-injected (Days 22–24 n = 12; Day 55–52 n = 12; Day 78–80 n = 12). Treatment groups are compared within each brain region. Asterisks denote significant differences in HACU rates. For all comparisons, a p<0.05 after Sidak’s correction for family-wise error was considered significant.

fCTX, STR, and HCC were dissected from NTB and TB mice on standard or 2% choline diet on Days 22–24 to assess the effects of a single injection of CYP+DOX; on Days 50–52 to assess the effects of 4 CYP+DOX injections on HACU; and on Days 78–80 to assess the late emergence, or persistence, of changes in HACU (Fig 6).

#### Multivariate analysis

Multivariate Analysis of Variance (MANOVA) identified a small but significant interaction between Tumor Status, Diet, and Chemotherapy on HACU [partial η^2^ = 0.05; F (3, 250) = 4.30, p<0.05]. Additionally, a moderate interaction between Diet, Chemotherapy, and Week of assessment [partial η^2^ = 0.09; F (3, 251) = 7.68, p<0.05] indicated notable changes in HACU across brain regions. To further understand these effects, we conducted univariate analyses within regions.

#### Univariate analyses

We performed analyses within each brain region to examine the specific effects of Diet, Chemotherapy, and Week on HACU in NTB and TB mice. In NTB mice, HACU remained unchanged across all brain regions after 1 (Day 22–24) or 4 (Day 50–52) weekly administrations of CYP+DOX, as well as 35 days post-treatment (Day 78–80), suggesting that HACU in NTB mice is not vulnerable to this dose combination.

Contrasting NTB mice, TB mice on a standard diet exhibited a significant decrease in HACU in the fCTX and STR after 4 weekly injections of CYP+DOX (Day 50–52) compared to saline-injected controls [fCTX: partial η^2^ = 0.04; F(1,125) = 5.14, p = 0.025; STR: partial η^2^ = 0.04; F(1,125) = 5.24, p = 0.024]. This reduction persisted in both regions when assessed 35 days later [fCTX: partial η^2^ = 0.03; F(1,252) = 4.25, p = 0.042; STR: partial η^2^ = 0.04; F(1,252) = 4.96, p = 0.028]. A delayed decrease in HACU was observed in the HCC of TB mice on a standard diet 35 days following their final injection of CYP+DOX [partial η^2^ = 0.03; F(1,252) = 4.37, p = 0.039] indicating both persistent and delayed effects of CYP+DOX on HACU.

Notably, 2% choline diet TB mice administered CYP+DOX showed no significant HACU changes in the fCTX, STR, or HCC at any time point compared to saline-injected controls, suggesting that increased dietary choline enhanced HACU. Furthermore, HACU was significantly higher in the STR and HCC of CYP+DOX injected mice TB mice on 2% choline diet compared to those on standard diet [STR: partial η^2^ = 0.07; F(1,125) = 9.63, p = 0.002; HCC: partial η^2^ = 0.09; F(1,125) = 12.64, p<0.001], indicating that a high choline diet may mask the attenuating effects of CYP+DOX on HACU.

## Discussion

The present study examined interactions between tumor status, AC chemotherapy, and dietary choline using an MMTv-PyVT mouse model of breast cancer. TB mice exhibited spatial memory deficits and alterations in HACU during and following CYP+DOX chemotherapy that were not present in NTB mice. The use of a choline-enriched diet mitigated the impairments in both HACU and spatial memory in TB mice, suggesting a protective role of choline against CRCIs. This study indicates that tumors themselves can contribute to vulnerability to CRCIs and underscores the potential use of a high choline diet in managing the physiological and cognitive side effects associated with cancer and its treatment.

### Tumors, Chemotherapy, Diet, and CRCIs

During treatment, CYP+DOX-treated TB mice exhibited a significantly reduced rate of tumor growth compared to the saline group, highlighting the efficacy of CYP+DOX chemotherapy in controlling tumor progression and indicating that the doses used were appropriate for a CRCI model. The overall tumor burden in the CYP+DOX group remained lower than saline-injected controls throughout the remainder of the study, indicating the long-term efficacy of this chemotherapy regimen in tumor control.

TB mice on a 2% choline diet displayed significant reductions in tumor growth compared to those on a standard diet suggesting an inhibitory effect of choline on tumor growth. This finding indicates that an intervention of increased dietary choline for cancer patients might play a role in modulating tumor growth and progression during treatment. Since abnormal choline metabolism is considered a hallmark of cancer [62] and choline can alter cell signaling via metabolite activity [63–65], perhaps increasing levels of circulating choline initiate changes that normalize choline metabolism. In addition, choline has been shown to regulate inflammation, decreasing proinflammatory cytokines [66–68], and an increase in some pro-inflammatory cytokines has been implicated in tumor cell proliferation, reduced apoptosis, and enhanced angiogenesis. Therefore, increasing circulating choline may influence tumor emergence and growth via these effects on cytokines. However, the role of these mechanisms in choline’s effects remains to be investigated.

Consistent with other studies, our results demonstrate that AC chemotherapy produces deficits in cognitive function [35, 69, 70]. This study utilized NTB and TB mice and CYP+DOX-associated deficits in spatial memory were observed solely in TB mice. We did not observe impaired MWM performance due to the presence of mammary tumors alone, similar to a prior study of Wistar rats with carcinogen induced mammary tumors [71], but only observed differences between NTB and TB mice following CYP+DOX injections. However, Schrepf et al (2010) did demonstrate poor spatial reference memory in the radial arm maze when compared to non-tumor bearing controls, and suggest that spatial working memory and reference memory may be compromised by the induction of neuroinflammatory pathways caused by presence of tumors alone [72]. Although some studies have demonstrated CRCIs in NTB animals, results are equivocal, due to differences in assessment paradigms [73], animal species or strain [34, 35, 69, 74, 75], and differences in chemotherapeutic agents and doses used. However, Flanigan et al. [76] assessed spatial memory in the MWM using non-tumor bearing C57Bl/6J mice, mice that are cross-bred with our MMTV-PyVT mice and failed to detect an effect of AC chemotherapy, consistent with our findings. Overall, these findings support an interpretation that tumors produce or enhance vulnerability to CRCIs and align well with clinical reports of deficits in cognitive function associated with the presence of tumors.

#### HACU in Response to Tumors, Chemotherapy, and Diet

Our findings have implications for understanding and potentially mitigating the cognitive side effects of chemotherapy. Our study is the first demonstration of the interplay between tumors, chemotherapy, and choline and their impact on HACU. HACU, the rate-limiting step for synthesizing ACh, was impaired in TB mice administered CYP+DOX. Given that spatial memory was impaired in groups that exhibited reduced HACU, but not those without HACU disruptions, our findings implicate reductions in HACU as a mechanism underlying CRCIs. The observation that a choline-enriched diet mitigates the changes in HACU induced by tumors and chemotherapy is especially significant given that increasing dietary choline also mitigated the emergence of impaired spatial memory. We and others have previously demonstrated that the administration of the acetylcholinesterase inhibitors donepezil or galantamine during chemotherapy can attenuate CRCIs [34, 53, 55]. The present findings suggest that these interventions may be effective in maintaining cholinergic function despite reduced HACU. Further, the present findings offer a non-pharmacological approach to support cognitive function and reinforce the importance of considering nutritional strategies to improve treatment outcomes.

### Impact of chemotherapy on ovarian function and estrogen levels

Our findings on proestrus suggest an impact of chemotherapy on ovarian function which aligns with other studies in both humans and animals. Given that the ovarian follicles are the primary source of E^2^, impaired ovarian function should result in a reduction of circulating E^2^ levels. In our study we observed impaired proestrus during chemotherapy, however, methodological limitations undermined our ability to confirm an effect on E^2^. Nevertheless, based on the observation that chemotherapy reduced proestrus frequency, reduced circulating E^2^ might contribute to alterations in HACU. However, further studies are necessary to develop a clear understanding of the role of estrogens in HACU changes and cognitive function in individuals receiving chemotherapy.

Although we observed a reduction in proestrus frequency caused by the introduction of a 2% choline diet, we did not observe reduced HACU in these mice. This makes it less straightforward to link impaired HACU to reductions in circulating E^2^, as indicated by a reduction in proestrus frequency. It is important to note that it is likely that since increasing choline availability compensates for reductions in HACU [32, 33] significant changes in HACU were not observed, despite reduced E^2^.

### Limitations and future research

Human studies indicate that tumors themselves may impair cognitive function and that tumors may interact with chemotherapy to produce cognitive impairments [31, 77, 78]. Indeed, the present study demonstrates a significant contribution of tumors to cognitive impairments associated with AC chemotherapy. Since many animal studies of CRCIs do not include tumor-bearing subjects, these studies may not accurately reflect the human condition and/or fail to identify consequences central to the symptomology of the cancer patient population exhibiting CRCIs. Therefore, future studies using animals to model CRCIs should include tumor-bearing animals to verify the findings of studies that utilized only non-tumor-bearing subjects.

The use of the MMTV-PyVT mouse model and CYP+DOX treatment regimen may limit the generalizability of the findings beyond breast cancer patients. Furthermore, the specific effects observed in our study need to be explored in diverse cancer types and treatment protocols beyond AC chemotherapy. In addition, specific tumor genotyping within the MMTV-PyVT mouse model should be performed, as the MMTV-PyVT breast cancer mouse model manifests multiple breast cancer tumor types [79].

While CYP+DOX effectively reduces tumor growth on its own, combining these drugs with a choline-enriched diet may offer additional benefits in managing tumor progression, suggesting a multifaceted approach to cancer therapy in which dietary modifications could complement pharmacological treatments to enhance overall treatment efficacy and potentially mitigate side effects. Future preclinical studies should explore the underlying mechanisms driving the mitigating effects of a choline-enriched diet on chemotherapy-induced cognitive impairments. This would include an examination of the effects of choline supplementation on ovarian follicles in tumor-bearing populations to examine whether the 2% choline diet-associated decrease in proestrus halts or slows the cell cycle of ovarian follicles, potentially providing a protective effect against CYP+DOX mediated ovarian ablation.

## Supporting information

S1 Fig Sankey Diagram of group allocation and attrition. The flow and distribution of animal numbers across different experimental groups, starting from Day 1 and continuing through critical points in the study protocol including the Morris Water Maze (MWM) and high-affinity choline uptake (HACU) assessments. The widths of the bands are proportional to the number of animals in each group, with NTB (non-tumor-bearing) and TB (tumor-bearing) mice differentiated by dietary (standard or 2% choline) and treatment (saline or CYP+DOX) categories. Attrition at various stages is depicted, including animals that did not continue due to poor baseline performance in the MWM, tumor size exceeding threshold criteria, or deaths due to unknown reasons, as well as data points lost due to video capture errors or sample processing errors.
(TIF)

S2 Fig Death probabilities of tumor-bearing mice across weeks of assessment. Each step in the curves denotes an event of death associated with tumor volume or unknown causes but does not include mice that were removed from the study due to planned tissue collection, poor baseline performance in the Morris water maze or experimental errors that precluded continuation (e.g. incorrect drug administration). After removal of mice from the analysis for events other than death or unknown causes the initial group sizes were as follows: TB STD Saline (n = 48); TB 2% Choline Saline (n = 38); TB STD DOX+CYP (n = 52); and TB 2% choline DOX+CYP (n = 41). The shaded red area indicates week 1 through week 4 of cyclophosphamide (CYP; 66.7 mg/kg, i.v.) and doxorubicin (DOX; 6.7 mg/kg, i.v.) or saline injections. Color references for groups are in the legend. TB = Tumor Bearing; STD = Standard Diet.
(TIF)

S3 Fig Comparative death probabilities of tumor-bearing mice across weeks of assessment. Each step in the curves denotes an event of death associated with tumor volume or unknown causes but does not include mice that were removed from the study due to planned tissue collection, poor baseline performance in the Morris water maze or experimental errors that precluded continuation (e.g. incorrect drug administration). The shaded red area indicates week 1 through week 4 of cyclophosphamide (CYP; 66.7 mg/kg, i.v.) and doxorubicin (DOX; 6.7 mg/kg, i.v.) or saline injection. Color references for groups are in the legend. *A)* compares tumor-bearing mice on a standard diet receiving saline (n = 48) versus those on a standard diet receiving CYP+DOX (n = 52); *B)* contrasts tumor-bearing mice on a 2% choline diet receiving saline injections (n = 38) with mice on a 2% choline diet receiving CYP+DOX (n = 41). *C)* compares tumor-bearing mice on a standard diet receiving saline injections to those on a 2% choline diet receiving saline injections; and D) compares tumor-bearing mice on a standard diet receiving CYP+DOX injections to those on 2% choline diet receiving CYP+DOX injections. TB = Tumor Bearing; STD = Standard Diet.
(TIF)

S4 Fig MWM training data. Legend indicates groups. Points represent group means; error bars represent +/- S.E.M. Non-tumor bearing (M-; n = 253) and tumor bearing (M+; n = 224) mice demonstrated a similar reduction of distance swam to locate the platform across platform trials indicating similar learning. The dashed line represents the maximum swim distance necessary to travel one full circle around the pool.
(TIF)

S1 File Raw data for HACU chemobrain manuscript. This file contains the raw data for the study.
(XLSX)

S2 File Bradford protein assay protocol. This is the Protocol used by this lab for Bradford Protein Assays.
(DOCX)

S3 File HACU assay protocol. This is the Protocol used by this lab to quantify HACU.
(DOCX)
