# Supplementary figures and images for: Cyclophosphamide- and doxorubicin-induced impairment of high affinity choline uptake and spatial memory can be prevented by dietary choline supplementation in breast tumor bearing mice
Source: PLoS One. 2024 Nov 21;19(11):e0305365. doi: 10.1371/journal.pone.0305365 (PMC11581227; doi:10.1371/journal.pone.0305365)

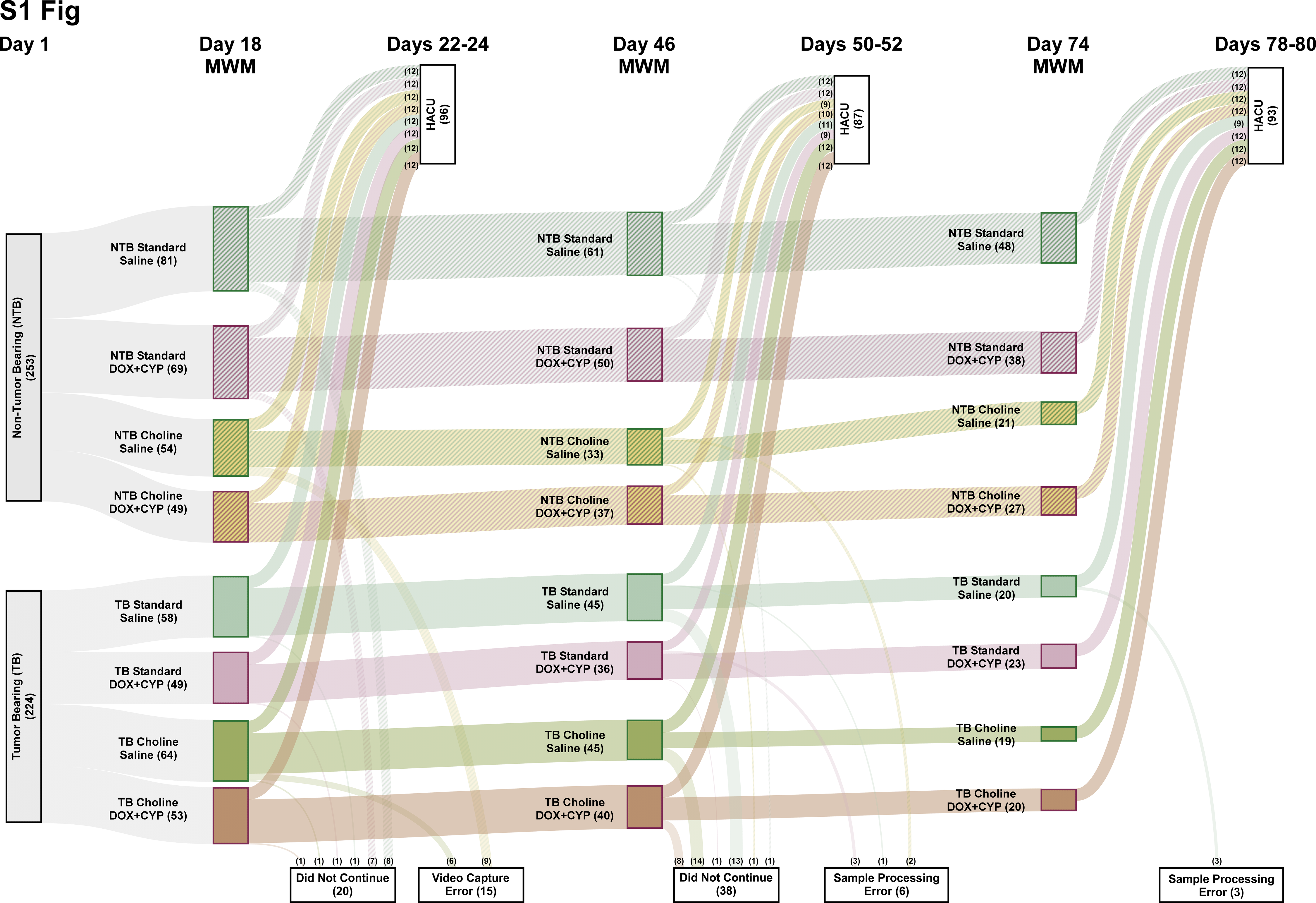

Supplement: S1 Fig — The flow and distribution of animal numbers across different experimental groups, starting from Day 1 and continuing through critical points in the study protocol including the Morris Water Maze (MWM) and high-affinity choline uptake (HACU) assessments. The widths of the bands are proportional to the number of animals in each group, with NTB (non-tumor-bearing) and TB (tumor-bearing) mice differentiated by dietary (standard or 2% choline) and treatment (saline or CYP+DOX) categories. Attrition at various stages is depicted, including animals that did not continue due to poor baseline performance in the MWM, tumor size exceeding threshold criteria, or deaths due to unknown reasons, as well as data points lost due to video capture errors or sample processing errors. (TIF) [file pone.0305365.s001.tif]

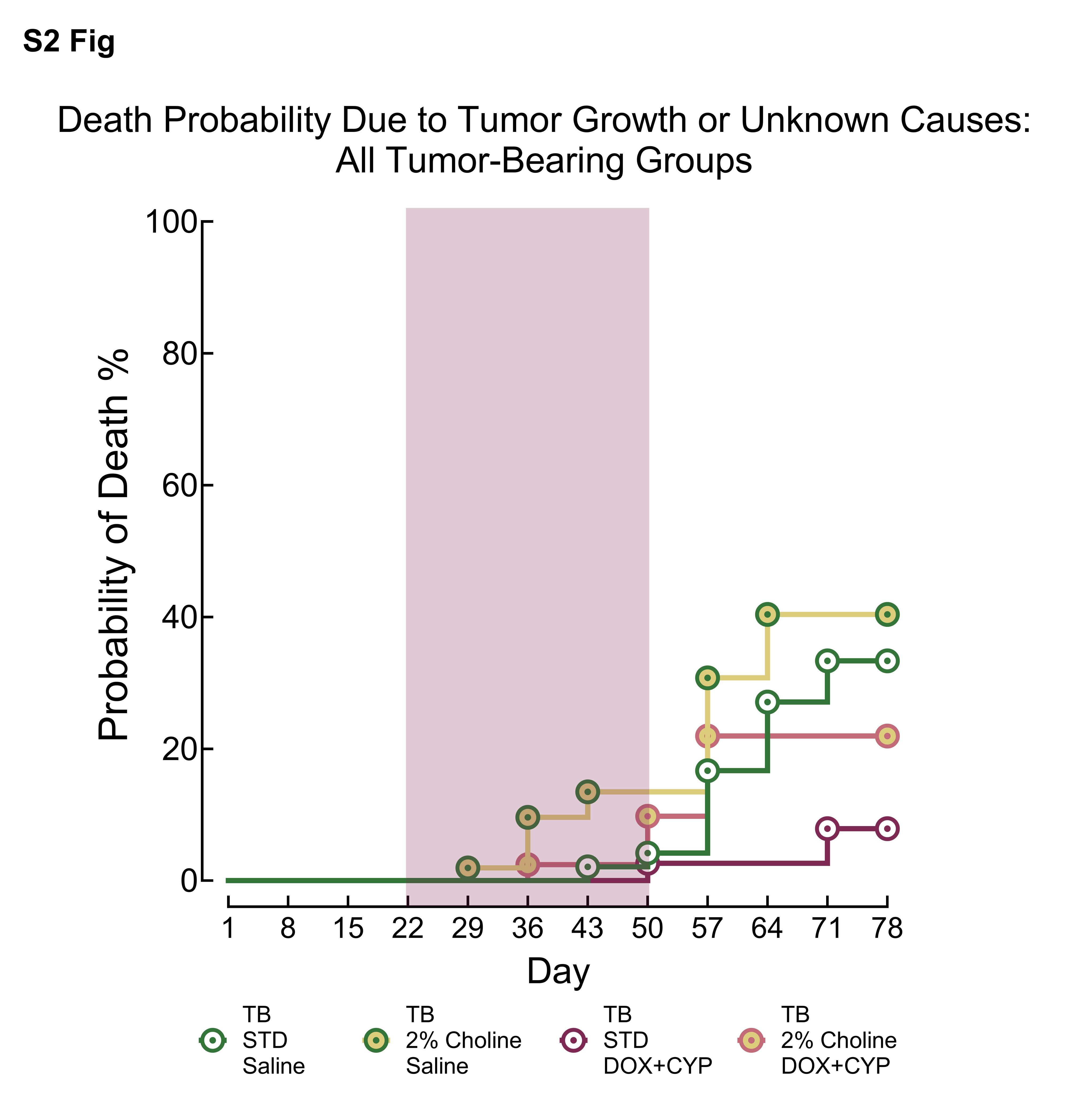

Supplement: S2 Fig — Each step in the curves denotes an event of death associated with tumor volume or unknown causes but does not include mice that were removed from the study due to planned tissue collection, poor baseline performance in the Morris water maze or experimental errors that precluded continuation (e.g. incorrect drug administration). After removal of mice from the analysis for events other than death or unknown causes the initial group sizes were as follows: TB STD Saline (n = 48); TB 2% Choline Saline (n = 38); TB STD DOX+CYP (n = 52); and TB 2% choline DOX+CYP (n = 41). The shaded red area indicates week 1 through week 4 of cyclophosphamide (CYP; 66.7 mg/kg, i.v.) and doxorubicin (DOX; 6.7 mg/kg, i.v.) or saline injections. Color references for groups are in the legend. TB = Tumor Bearing; STD = Standard Diet. (TIF) [file pone.0305365.s002.tif]

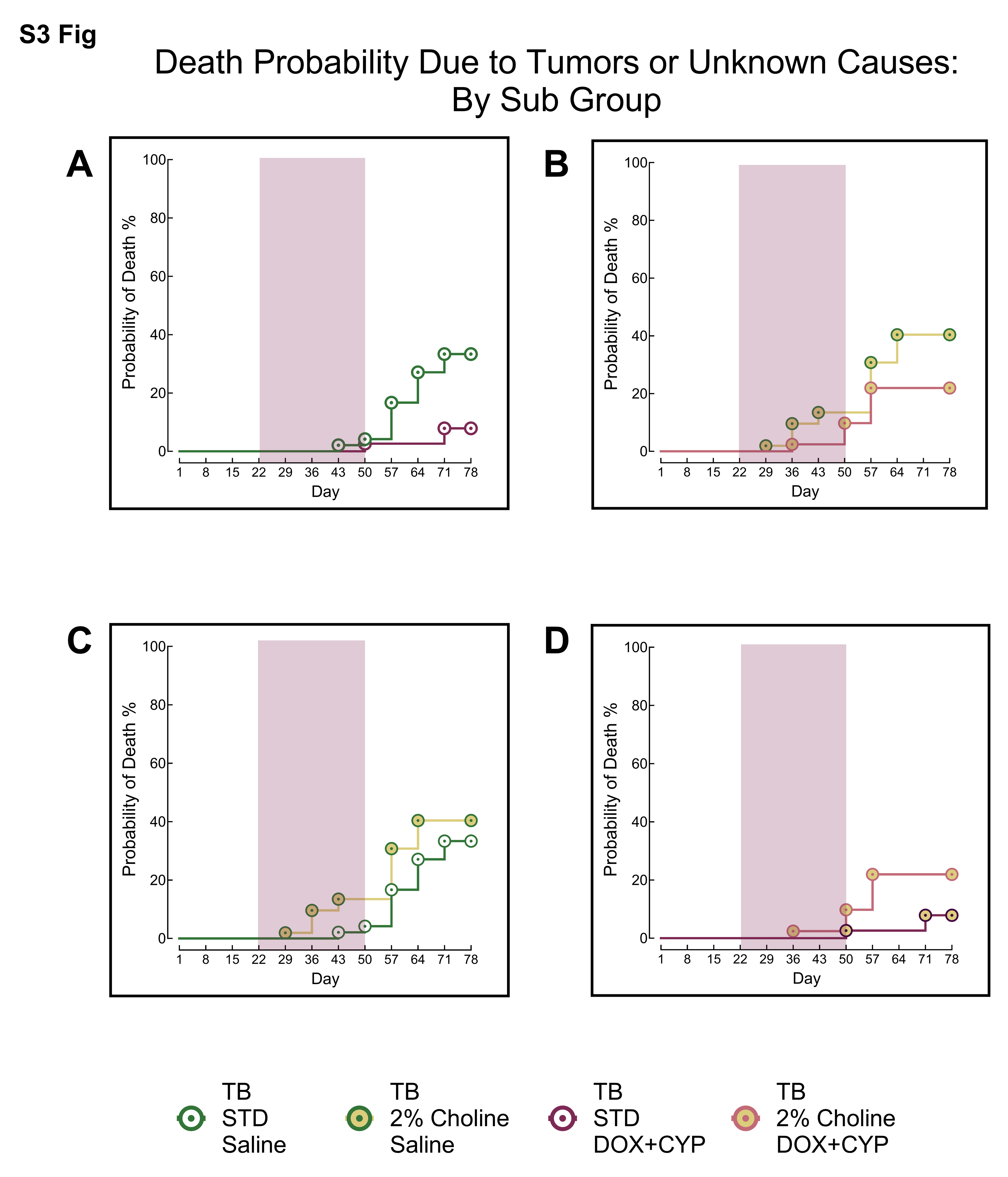

Supplement: S3 Fig — Each step in the curves denotes an event of death associated with tumor volume or unknown causes but does not include mice that were removed from the study due to planned tissue collection, poor baseline performance in the Morris water maze or experimental errors that precluded continuation (e.g. incorrect drug administration). The shaded red area indicates week 1 through week 4 of cyclophosphamide (CYP; 66.7 mg/kg, i.v.) and doxorubicin (DOX; 6.7 mg/kg, i.v.) or saline injection. Color references for groups are in the legend. A) compares tumor-bearing mice on a standard diet receiving saline (n = 48) versus those on a standard diet receiving CYP+DOX (n = 52); B) contrasts tumor-bearing mice on a 2% choline diet receiving saline injections (n = 38) with mice on a 2% choline diet receiving CYP+DOX (n = 41). C) compares tumor-bearing mice on a standard diet receiving saline injections to those on a 2% choline diet receiving saline injections; and D) compares tumor-bearing mice on a standard diet receiving CYP+DOX injections to those on 2% choline diet receiving CYP+DOX injections. TB = Tumor Bearing; STD = Standard Diet. (TIF) [file pone.0305365.s003.tif]

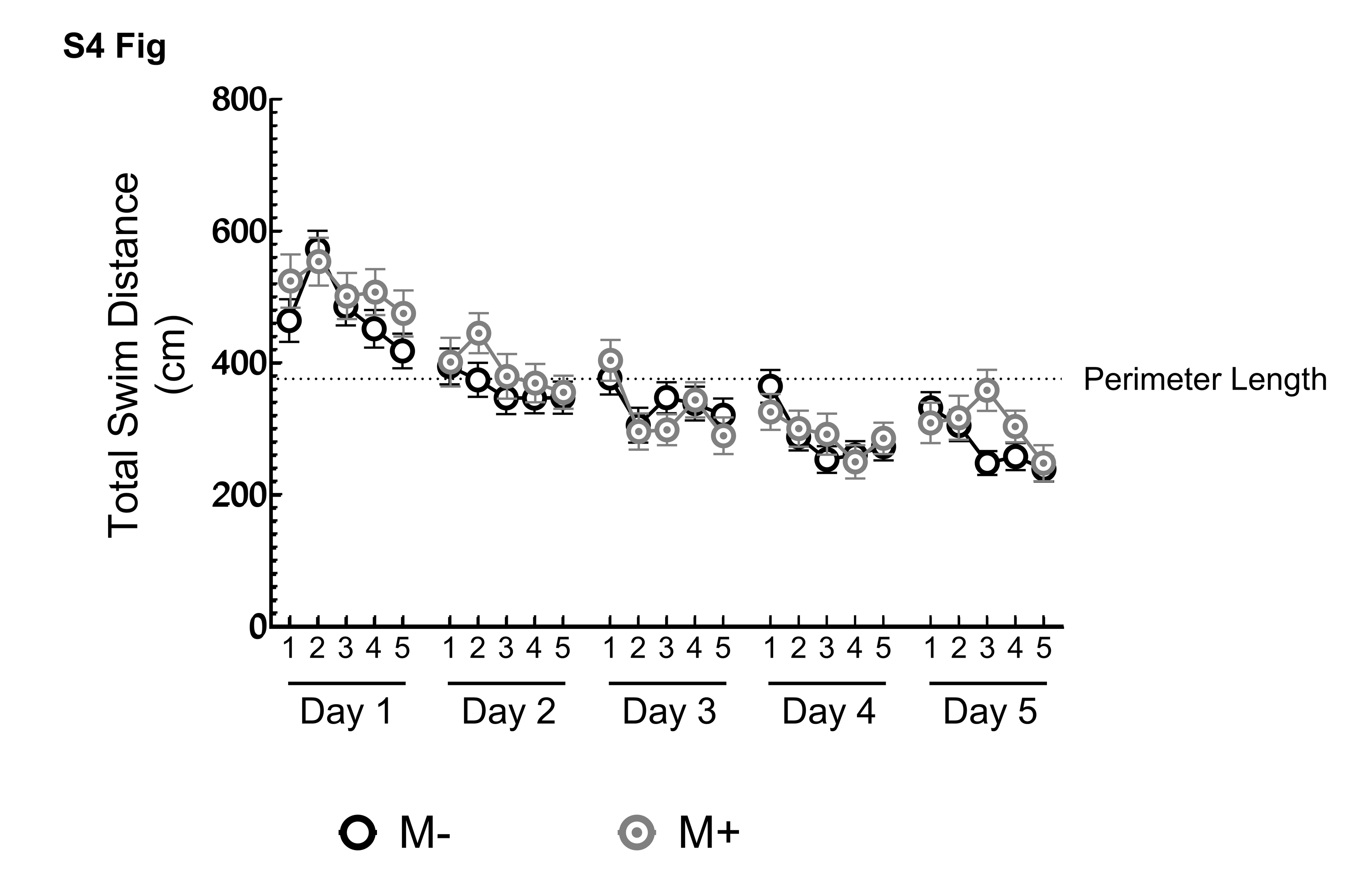

Supplement: S4 Fig — Legend indicates groups. Points represent group means; error bars represent +/- S.E.M. Non-tumor bearing (M-; n = 253) and tumor bearing (M+; n = 224) mice demonstrated a similar reduction of distance swam to locate the platform across platform trials indicating similar learning. The dashed line represents the maximum swim distance necessary to travel one full circle around the pool. (TIF) [file pone.0305365.s004.tif]
