# Supplementary material for: Cyclophosphamide- and doxorubicin-induced impairment of high affinity choline uptake and spatial memory can be prevented by dietary choline supplementation in breast tumor bearing mice
Source: PLoS One. 2024 Nov 21;19(11):e0305365. doi: 10.1371/journal.pone.0305365 (PMC11581227; doi:10.1371/journal.pone.0305365)
Supplement: S2 File — This is the Protocol used by this lab for Bradford Protein Assays. (DOCX) [file pone.0305365.s006.docx]

Bradford (Coomassie) Protein Assay Protocol, updated 3/6/2024:

Materials:
 Thermo Scientific Coomassie Protein Assay Kit (#23200)
 100 ml 2N NaOH:

-add 8g NaOH to 100 ml de-ionized water

-stir on stir plate for 30 minutes or until dissolved

-pour into properly labeled container (contents, date, preparer)

-contents can be stored at room temperature for up to one year

Kreb’s Ringers HEPES Buffer:

|  |  | **STOCKS** |  |  |  |  |  |  | **for 250ml** | **for 500ml** |
| --- | --- | --- | --- | --- | --- | --- | --- | --- | --- | --- |
|  |  | **Compound** | **Mass (g/mol)** | **mg/ml** | **Molarity** |  |  |  | **grams** | **grams** |
|  |  | NaCl | 58.44 | 144.9312 | 2.48 |  |  |  | 36.2328 | 72.4656 |
|  |  | KCl | 74.56 | 14.9120 | 0.20 |  |  |  | 3.7280 | 7.4560 |
|  |  | CaCl2 | 111.00 | 11.1000 | 0.10 |  |  |  | 2.7750 | 5.5500 |
|  |  | MgSO4 | 120.36 | 12.0361 | 0.10 |  |  |  | 3.0090 | 6.0181 |
|  |  | HEPES-NaOH | 238.30 | 95.3208 | 0.40 | pH to 7.4 w NaOH | |  | 23.8302 | 47.6604 |
|  |  |  |  |  |  |  |  |  |  |  |
|  |  |  |  |  |  |  |  |  |  |  |
|  |  | **1L Krebs Ringer's HEPES Buffer** |  |  | **Final** |  |  |  | **for 250ml** | **for 500ml** |
|  | **ADD** | **Stock Solution** | **Molarity** | **Volume (ml)** | **Molarity** |  |  |  | **Volume (ml)** | **Volume (ml)** |
|  |  | NaCl | 2.48 | 50 | 0.1240 |  |  |  | 12.5000 | 25.0000 |
|  |  | KCl | 0.20 | 25 | 0.0050 |  |  |  | 6.2500 | 12.5000 |
|  |  | CaCl2 | 0.10 | 15 | 0.0015 |  |  |  | 3.7500 | 7.5000 |
|  |  | MgSO4 | 0.10 | 13 | 0.0013 |  |  |  | 3.2500 | 6.5000 |
|  |  | HEPES-NaOH | 0.40 | 50 | 0.0200 |  |  |  | 12.5000 | 25.0000 |
|  |  |  |  |  |  |  |  |  |  |  |
|  | **ADD** | **Compound** | **Mass (g/mol)** | **g/L** | **Molarity** |  |  |  | **grams** | **grams** |
|  |  | Glucose | 180.16 | 1.8016 | 0.0100 |  |  |  | 0.4504 | 0.9008 |
|  |  |  |  |  |  |  |  |  |  |  |
|  | **QS TO 1L WITH ddH2O** | | |  |  |  |  |  |  |  |

Flat-bottom, non-treated 96 well plate (CytoOne #CC7672-7596)

Protocol:

1. Set out Coomassie solution to reach room temperature, making sure the solution is protected from light
2. Make dilution cascade for albumin (BSA) standards:
   1. Label 8x 1.5 ml Eppendorf tubes from A-H, then use the following table to prepare standard solutions, starting with filling vials with the appropriate volume of Krebs-ringer HEPES buffer, vortexing vials for 5 seconds with each addition of BSA:

| Vial | Volume Krebs-ringer HEPES buffer | Volume and Source of BSA | Final BSA concentration |
| --- | --- | --- | --- |
| A | 0uL | 300uL stock | 10ug/5uL |
| B | 125uL | 375uL stock | 7.5ug/uL |
| C | 300uL | 300uL stock | 5ug/uL |
| D | 175uL | 175uL vial B | 3.75ug/uL |
| E | 325uL | 325uL vial C | 2.5ug/uL |
| F | 325uL | 325uL vial E | 1.25ug/uL |
| G | 325uL | 325uL vial F | 0.625ug/uL |
| H | 400uL | 0uL | 0.0ug/uL (background) |

1. Sample preparation:
   1. Aliquot 10uL of samples into labeled 1.5mL Eppendorf tubes
   2. Add 10uL of 2N NaOH to each labeled sample tube (NOT standards)
   3. Vortex each sample tube for 5s
2. Use a 96-well plate layout template to designate assay standards and samples to wells.
   1. Run all standards and samples in triplicate, using sample layout shown below as a guide:

| A | A | A | S1 | S1 | S1 | S9 | S9 | S9 | S17 | S17 | S17 |
| --- | --- | --- | --- | --- | --- | --- | --- | --- | --- | --- | --- |
| B | B | B | S2 | S2 | S2 | S10 | S10 | S10 | S18 | S18 | S18 |
| C | C | C | S3 | S3 | S3 | S11 | S11 | S11 | S19 | S19 | S19 |
| D | D | D | S4 | S4 | S4 | S12 | S12 | S12 | S20 | S20 | S20 |
| E | E | E | S5 | S5 | S5 | S13 | S13 | S13 | S21 | S21 | S21 |
| F | F | F | S6 | S6 | S6 | S14 | S14 | S14 | S22 | S22 | S22 |
| G | G | G | S7 | S7 | S7 | S15 | S15 | S15 | S23 | S23 | S23 |
| H | H | H | S8 | S8 | S8 | S16 | S16 | S16 | S24 | S24 | S24 |

1. Pipette 5uL of each standard and sample into previously designated wells
2. Add 250uL of Coomassie reagent to each well with repeat pipette
3. Mix on plate mixer for 30 seconds at 300 rpm
4. Incubate plate at room temperature (without shaking) for 10 minutes
5. Measure absorbance at ≈595nm on plate reader (instructions for plate reader setup found with/varies by manufacturer)
6. Important to note: sample and standard solutions are measured in ug/uL. (Multiply by .4 to convert results to mg for eventual pg/mg reporting)
