# Supplementary material for: Cyclophosphamide- and doxorubicin-induced impairment of high affinity choline uptake and spatial memory can be prevented by dietary choline supplementation in breast tumor bearing mice
Source: PLoS One. 2024 Nov 21;19(11):e0305365. doi: 10.1371/journal.pone.0305365 (PMC11581227; doi:10.1371/journal.pone.0305365)
Supplement: S3 File — This is the Protocol used by this lab to quantify HACU. (DOCX) [file pone.0305365.s007.docx]

24 hours prior to preparation of [3H] Choline from stock

1. Hydrate SP-Sephadex (Sigma SP-C25-120) by putting some in a 20ml scintillation vial and adding ddH2O (sephadex will double in volume when hydrated).
2. Swirl gently, do not shake.
3. Let sit overnight and store in refrigerator.

Preparation of [3H] Choline Chloride from stock

1. Use BioRad disposable column (cat# 731-1550) and form a 1ml resin bed with sephadex.
2. Wash 2x with 1ml ddH2O.
3. Load with 160uL [3H] choline stock from New England Nuclear (NET109001MC; 1 mCi/ml ethanol).
   1. *The sephadex is non-polar, hydrophilic and lipophilic and will slow the passage of [3H] choline.*
4. Wash column with 10mL water and discard in radioactive liquid waste.
   1. *The [3H] choline will remain in the sephadex while impurities are washed out.*
5. Add 0.5mL of 300mM NaCl (17.532 mg/ml) and discard run off in radioactive waste.
   1. *[3H] choline chloride will desorb off the sephadex and dissolve in the NaCl.*
6. Put a 20mL scintillation vial under the column and collect [3H] choline chloride by washing the column with 2mL of 300mM NaCl.
7. This should yield a final concentration of approximately 80uCi/mL.
8. Test final concentration on scintillation counter.

**Prepare the day before:**

1. Glass homogenization tubes labelled with animal # and brain region
   1. 1 tube per brain region per animal
2. 1.5mL microfuge tubes labelled “P1” with animal #, brain region, date
   1. 1 tube per brain region per animal
3. 1.5mL microfuge tubes labelled “P2” with animal #, brain region, date
   1. 1 tube per brain region per animal
4. 0.7mL microfuge tubes labelled “P2 Protein” with animal #, brain region, date
   1. 3 tubes per brain region per animal
5. Incubation test tubes labelled “Hot” or “Cold” with animal #, brain region, date
   1. 2 tubes per brain region per animal
6. 20mL Scintillation vials labelled “Hot” or “Cold” with animal #, brain region, date
   1. 2 vials per brain region per animal
7. 0.32M sucrose (54.77gm/500mL ddH2O)
8. Krebs-Ringers HEPES Buffer

|  | **STOCKS** |  |  |  |  |  |  | **for 250ml** | **for 500ml** |
| --- | --- | --- | --- | --- | --- | --- | --- | --- | --- |
|  | **Compound** | **Mass (g/mol)** | **mg/ml** | **Molarity** |  |  |  | **grams** | **grams** |
|  | NaCl | 58.44 | 144.9312 | 2.48 |  |  |  | 36.2328 | 72.4656 |
|  | KCl | 74.56 | 14.9120 | 0.20 |  |  |  | 3.7280 | 7.4560 |
|  | CaCl2 | 111.00 | 11.1000 | 0.10 |  |  |  | 2.7750 | 5.5500 |
|  | MgSO4 | 120.36 | 12.0361 | 0.10 |  |  |  | 3.0090 | 6.0181 |
|  | HEPES-NaOH | 238.30 | 95.3208 | 0.40 | pH to 7.4 w NaOH | |  | 23.8302 | 47.6604 |
|  |  |  |  |  |  |  |  |  |  |
|  |  |  |  |  |  |  |  |  |  |
|  | **1L Krebs Ringer's HEPES Buffer** |  |  | **Final** |  |  |  | **for 250ml** | **for 500ml** |
| **ADD** | **Stock Solution** | **Molarity** | **Volume (ml)** | **Molarity** |  |  |  | **Volume (ml)** | **Volume (ml)** |
|  | NaCl | 2.48 | 50 | 0.1240 |  |  |  | 12.5000 | 25.0000 |
|  | KCl | 0.20 | 25 | 0.0050 |  |  |  | 6.2500 | 12.5000 |
|  | CaCl2 | 0.10 | 15 | 0.0015 |  |  |  | 3.7500 | 7.5000 |
|  | MgSO4 | 0.10 | 13 | 0.0013 |  |  |  | 3.2500 | 6.5000 |
|  | HEPES-NaOH | 0.40 | 50 | 0.0200 |  |  |  | 12.5000 | 25.0000 |
|  |  |  |  |  |  |  |  |  |  |
| **ADD** | **Compound** | **Mass (g/mol)** | **g/L** | **Molarity** |  |  |  | **grams** | **grams** |
|  | Glucose | 180.16 | 1.8016 | 0.0100 |  |  |  | 0.4504 | 0.9008 |
|  |  |  |  |  |  |  |  |  |  |
| **TOP OFF TO 1L WITH ddH2O** | |  |  |  |  |  |  |  |  |

Prepare Hot/Cold Choline Solution the morning of using washed [3H] Choline Chloride

- 1. Determine volume needed for 0.5uCi [3H] Choline/tube
     1. Ex. If washed [3H] Choline Chloride is 80uCi/mL then 6.25uL = 0.5nCi [3H] Choline.
     2. Use the excel spreadsheet below to calculate necessary fluid amounts.

Dissections for P1 and P2 samples

1. Bring to Vivarium
   1. Homogenization tubes labelled for each animal and brain region to be collected
   2. Ziploc bags labelled with animal #s and date
   3. Blood collection vials labelled with animal #s and date
   4. 0.32M Sucrose
   5. 2 Ice Buckets
   6. Ice for both buckets
   7. 1mm sectioning block
   8. Razor blades
   9. 50ml Beaker
   10. Ceramic plate
   11. 0.2m Filter Paper
   12. Weigh paper
   13. Biohazard bags
   14. Small Funnel
   15. 2 pair of Scissors (for skin and for skull)
   16. Forceps
   17. 2 Dull edge drug spatulas
2. Should be at Vivarium
   1. Guillotine
   2. Scale (capable of measuring in the 1-10mg range accurately)
3. Fill a bucket with ice and place a ceramic dish upside down on the surface, creating a chilled surface for dissections.
4. Chill surgical instruments and agents:
   1. 0.32M Sucrose in a 50mL beaker for chilling the brain.
   2. Scissors for cutting the skin.
   3. Scissors for cutting thought the skull (this will dull these scissors).
   4. Rongers, or Forceps strong enough for the removal of skull sections.
   5. 2 dull edged drug spatuala for teasing out brain sections and collecting samples.
5. Fill each homogenization tube with 1mL 0.32 sucrose and put in ice to chill.
6. Have the scale in arms reach of the dissection area. Set to the side prepared weigh paper.
7. Have small 0.2m filter papers available to use as a work surface.
8. Place guillotine by the work sink with a biohazard bag in the sink to collect the body.
   1. If sacrificing a tumor animal, have a small, labelled Ziploc bag available to save the body for dissection of tumors.
9. Place blood collection vial in the sink with a funnel attached ready to receive the body.
10. Rapidly decapitate the animal, place the body in the blood collection vial funnel.
11. Place filter paper on top of the dry ceramic dish and place the head on top of the filter paper.
    1. Cut back the skin from the base of the head to the tip of the nose and fold to either side of the head.
    2. Place the tip of the skull scissors underneath the base of the skull, pointing the tip toward the skull.
    3. Push upward against the skull, away from the brain, as you cut through the skull from back to front.
    4. Use forceps to pull pieces of the skull away from the brain.
       1. Do not take big pieces of skull as large pieces can damage the brain as you pull them out.
    5. Grab the flaps of skin of the head from underneath the skull and turn the head upside down.
    6. Hold the head above the small beaker containing chilled 0.32M sucrose.
    7. Use a blunt spatula to slide underneath the brain from the base and tease the tissue away from the skull base moving from the back of the head toward the nose until the brain falls into the sucrose. There are several cranial nerves that will need to be severed or broken for the brain to separate from the skull.
12. Let the brain chill for 5min.
    1. During this time, clean the guillotine area, collect your blood sample and place on ice. Prepare for the next brain removal.
13. Remove the chilled brain from the 0.32M sucrose, dry the surface and place in the 1mm sectioning block.
14. Use 5 razor blades to section the brain. Be careful not to allow the brain to shift while cutting through the tissue.
15. Dry the ceramic dish.
16. Place each tissue section flat on the chilled surface of the ceramic dish.
17. As quickly as possible, isolate the first specific brain region of interest (ROI) from the brain sections.
18. Weigh the ROI.
19. Place the ROI in the correctly labelled homogenization tube. Make sure the tissue is completely submerged.
20. Bring chilled tubes with tissue back to wet lab for processing.

Preparation of P1 and P2 samples

1. Homogenize each tissue sample on ice with 7 slow, complete strokes @ 500rpm w glass:Teflon homogenizer.
2. Place back in ice bucket.
3. Transfer homogenate to 1.5mL microfuge tube labelled “P1” and put on ice.
   1. Centrifuge homogenate @4⁰C, 1160g for 10min.
4. Take 900uL of supernatant (or as much supernatant as is available) without taking any pellet and transfer to centrifuge tube labelled “P2” and put on ice.
   1. Centrifuge supernatant @4⁰C, 20,800g for 20min.
5. Pour off supernatant and resuspend pellet in 0.32 sucrose.
   1. mL of sucrose = original wet weight (mg)/80.
   2. resuspend using manual 1mL pipette.
6. Aliquot 3 10uL P2 samples for protein assays.

Choline Uptake Assay

1. Set up 2 water baths:
   1. 1 at 37⁰C with test tube trays with the bottom 1/5^th^ beneath the water line.
   2. 1 at 0⁰C (ice slurry).
2. Pre-wet Millipore filter (cat# HAWP 02500 0.45uM pore size) by soaking in distilled water 15-30min prior to use.
   1. Load onto Millipore filtration apparatus 2min before use.
3. Place 0.8mL Krebs-Ringers HEPES buffer into iced test tubes.
   1. Add 0.1mL of Hot/Cold Choline containing 1nmol of choline and 0.5uCi of [3H] Choline to all tubes.
   2. Vortex.
4. Note: The filter apparatus only holds 12 filters, enough to assay 6 samples at a time. If you plan to run more than 6 samples you will need to run in batches or use a second apparatus AND have an assistant who can terminate the assay @5min while you continue to load P2 homogenate.
5. Before starting reaction have a reliable timer ready an begin counting up when the first P2 homogenate is vortexed.
6. To start reaction add 0.1ml of P2 homogenate to buffer/choline mixture in test tubes. Add P2 1 tube at a time.
   1. Add 0.1mL of the 1^st^ P2 homogenate, Vortex and place in 37⁰C water bath.
   2. Wait 20secs
   3. Add 0.1mL of P2 homogenate from SAME P2 homogenate, vortex and place in 0⁰C water bath.
   4. Wait 20secs
   5. Add 0.1mL of the 2^nd^ P2 homogenate, Vortex and place in 37⁰C water bath.
   6. Wait 20secs
   7. Add 0.1mL of P2 homogenate from SAME P2 homogenate, vortex and place in 0⁰C water bath.
   8. Wait 20secs
   9. CONTINUE for the 3^rd^, 4^th^, 5^th^ and 6^th^ homogenate.
7. Verify that all wells of Millipore filtration have wet filters and that the wells are plugged.
   1. Turn on vacuum.
8. At 5min (300sec) remove 1^st^ P2 sample from 37⁰C water bath and pour over pre-wet Millipore filter.
   1. Rinse tube 3x with 2mLs ice cold Krebs-Ringers HEPES buffer and pour rinse over sample/filter.
   2. Dispose of tube in solid radioactive waste.
9. At 5min 20sec (320sec) remove 1^st^ P2 sample from 0⁰C water bath and pour over pre-wet Millipore filter.
   1. Rinse tube 3x with 2mLs ice cold Krebs-Ringers HEPES buffer and pour rinse over sample/filter.
   2. Dispose of tube in solid radioactive waste.
10. CONTINUE every 20sec for 2^nd^, 3^rd^, 4^th^, 5^th^ and 6^th^ sample pairs.
11. Transfer filters to wide mouth 20mL scintillation vials and add 1mL ethylene glycol monoethyl ether (Fisher cat# E180-1)
    1. Shake on shaker until filers are dissolved (15-30min).
12. Add 10mL Ecolume, vortex and count an scintillation counter (10min/sample).
